# LCN2 induces neuronal loss and facilitates sepsis-associated cognitive impairments

**DOI:** 10.1038/s41419-025-07469-4

**Published:** 2025-03-01

**Authors:** Cuiping Guo, Wensheng Li, Yi Liu, Abdoul Razak Mahaman Yacoubou, Jianzhi Wang, Rong Liu, Shusheng Li, Xiaochuan Wang

**Affiliations:** 1 Department of Emergency Medicine & Department of Critical Care Medicine, Tongji Hospital, Tongji Medical College, Huazhong University of Science and Technology, Wuhan, China; 2 Institute of Biomedical Sciences, School of Medicine, Hubei Key Laboratory of Cognitive and Affective Disorders, Jianghan University, Wuhan, China; 3 Department of Pathophysiology, School of Basic Medicine, Key Laboratory of Education Ministry/Hubei Province of China for Neurological Disorders, Tongji Medical College, Huazhong University of Science and Technology, Wuhan, China; 4 Co-innovation Center of Neuroregeneration, Nantong University, Nantong, Jiangsu China

**Keywords:** RNAi, Neurological disorders

## Abstract

Sepsis-associated encephalopathy (SAE) is a severe neurological syndrome marked by widespread brain dysfunctions due to sepsis. Despite increasing data supporting the hypothesis of neuronal damage, the exact mechanism of sepsis-related cognitive disorders and therapeutic strategies remain unclear and need further investigation. In this study, a sepsis model was established in C57 mice using lipopolysaccharide (LPS). The findings demonstrated that LPS exposure induced neuronal loss, synaptic and cognitive deficits accompanied by mitochondrial damage. Bioinformatics and western blot analyses demonstrated a significant increase in Lipocalin-2 (LCN2) during sepsis as a key hub gene involved in immune and neurological inflammation. Interestingly, the recombinant LCN2 protein exhibited similar effects on synaptic dysfunction and cognitive deficits in C57 mice. Conversely, downregulating LCN2 effectively nullified the impact of LPS, leading to the amelioration of synaptic and cognitive deficits, neuronal loss, and reactive oxygen species (ROS)-associated mitochondrial damage. These findings suggest a novel etiopathogenic mechanism of SAE, which is initiated by the increased LCN2, leading to neuronal loss and cognitive deficit. Inhibition of LCN2 could be therapeutically beneficial in treating sepsis-induced synaptic and cognitive impairments.

## Introduction

Sepsis-induced systemic inflammation can lead to acute cerebral dysfunction, manifesting as delirium, coma, and cognitive dysfunction [1–3]. The pathophysiology of sepsis-associated encephalopathy is complex and involves multiple mechanisms that collectively contribute to brain dysfunction and injury. Despite growing interest in the hypothesis of neuronal damage, the precise underpinning mechanism and therapeutic strategies remain unclear, highlighting the need to integrate relevant molecular mechanisms and develop more effective treatments based on a deeper understanding of this mechanistic SAE pathophysiology.

Astrocytes play pivotal roles in driving inflammatory brain injury due to their critical involvement in brain-immune interfaces [4, 5]. They are reported to orchestrate the effects of immune cells in the central nervous system (CNS) by acting as a surveillance and integration center for inflammatory signals [6–8]. However, under septic exposure, dysregulation of astrocytes in cellular functions, including the regulation of neuronal activity, neurotransmitter release, and synaptic pruning, has been implicated in neuropsychiatric disorders [9]. The role of lipocalin-2 (LCN2) in the peripheral system is well-established. Recent findings indicate a connection between LCN2 and central nervous system damage. LCN2 plays biological roles in neurons, microglia, astrocytes, and endothelial cells. It acts as an acute phase protein after neuroinflammation [10–12]. In the CNS, astrocytes are the main source of LCN2 during neuroinflammation. Previous studies have demonstrated that LCN2 regulates pathological mechanisms such as cell death, neuroinflammation, blood-brain barrier disruption, oxidative stress, iron dysregulation, and neurovascular unit dysfunction [13–16]. Meanwhile, the levels of LCN2 in learning and memory-related hippocampus are markedly increased during sepsis [17, 18]. However, it is unclear whether LCN2-associated cell death is involved in the pathogenesis of SAE.

Overactivation of astroglia is involved in the progression of brain dysfunction by deteriorating the blood-brain barrier (BBB) and enhancing the release of cytokine [19, 20]. Whereas their inhibition is beneficial in reducing the brain oxidative damage and inflammation and improving cognitive function in sepsis [21–23]. Accumulating evidence underscores the relationship between mitochondrial dysfunction and disrupted neuroinflammatory pathways in the pathophysiology of SAE. Mitochondria are double-membrane organelles that produce energy and serve as the main site of aerobic respiration in the cell, including the synthesis of adenosine triphosphate (ATP), production and clearance of reactive oxygen species (ROS) [24–27]. The pathogenesis of SAE has increasingly been linked with mitochondrial dysfunction. Emerging evidence from these observations supports the significance of the neuronal apoptosis signaling pathway in the context of SAE.

Here, we demonstrated that mice with sepsis exhibit neuronal loss, which mediates sepsis-related synaptic and cognitive deficits accompanied by LCN2 upregulation. Additionally, downregulation of LCN2 in sepsis mice attenuated neuronal loss, synaptic damage, and cognitive impairments probably by restoring mitochondrial function. Our findings therefore provide new insights into the pathogenesis of SAE and provide a basis for potential therapeutic strategies.

## Results

### LPS-induced cognitive impairments, accompanied by a significant upregulation of LCN2

LPS is a unique component of gram-negative bacteria cell walls and has been widely used as a research tool that can trigger a cascade of immune responses and toxic pathophysiological activities, releasing endotoxins and causing inflammation and sepsis phenotype [28–30]. In the current study, the LPS-induced sepsis mouse model is used to explore the effects of sepsis on cognitive function. Open-field test data indicated no significant difference in the total distance covered between the LPS and control groups (sFig. 1A). Interestingly, the Novel Object Recognition (NOR) test indicated that the novel object preference was significantly reduced following LPS treatment (sFig. 1B). In addition, the Y-maze test, assessing memory and learning abilities, revealed that LPS mice exhibited significantly lesser duration spent (sFig. 1C) and fewer crossings (sFig. 1D) in the novel arm compared to the control group. We utilized ELISA kits to detect inflammatory cytokines. The findings revealed that LPS significantly increased the levels of inflammatory cytokines, including IL-1β (sFig. 1E) and IL-6 (sFig. 1F), which can trigger acute inflammatory reaction.

**Fig. 1:**
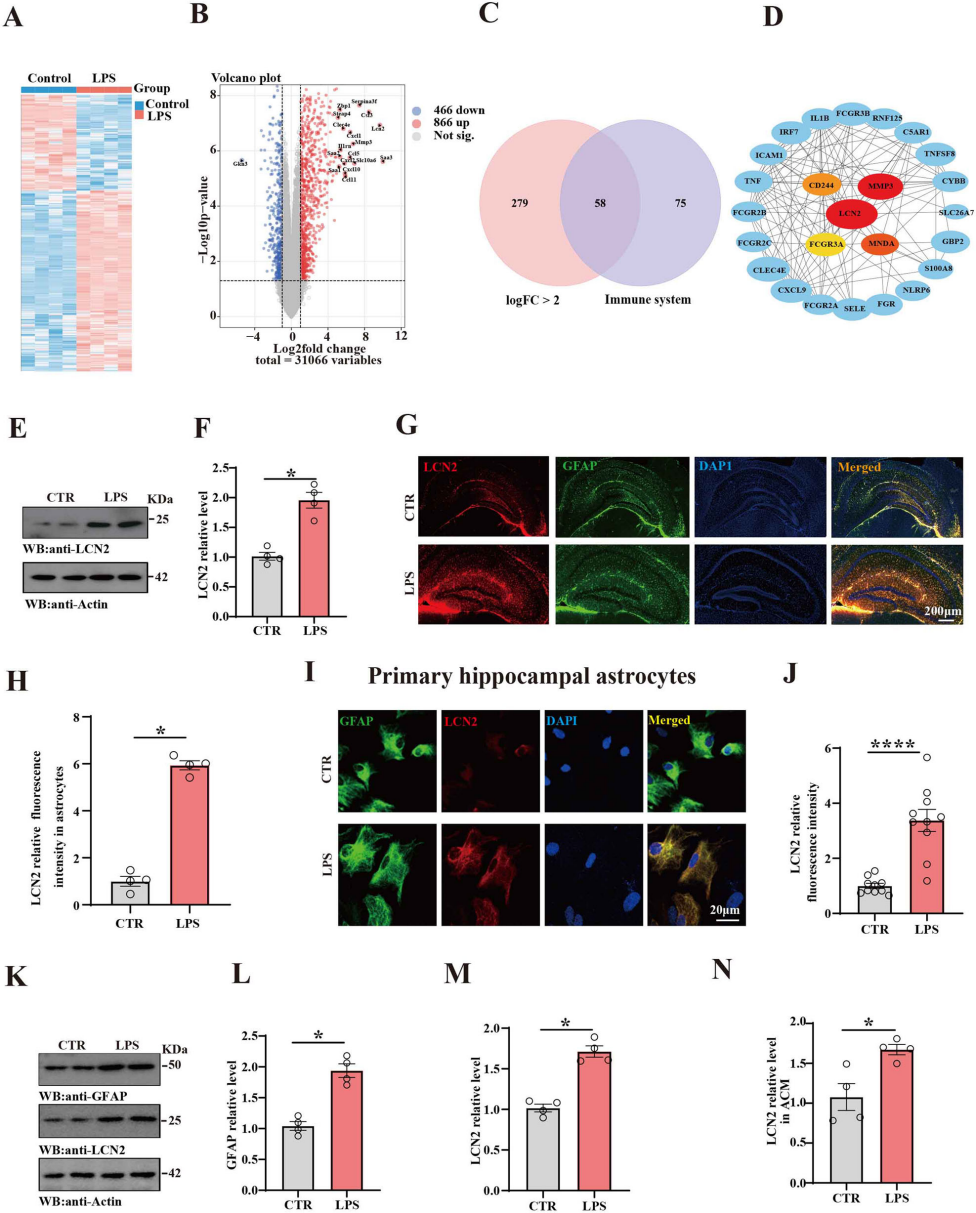
LPS-induced significant upregulation of LCN2. **A** Heatmap illustrating the expression levels of differentially expressed genes (DEGs) identified in dataset GSE88959. **B** Volcano plot of DEGs in GSE88959. Genes with |Log2FoldChange| > 5 are highlighted. **C** Venn diagram illustrates the intersection between DEGs (|Log2FoldChange| > 2 and P < 0.05) and core enrichment genes in the “Immune system” pathways (Reactome). **D** Protein-Protein Interaction (PPI) network of intersected genes, highlighting LCN2, CD244, MMP3, FCGR3A, and MNDA as hub genes. Size represents Log2FoldChange. **E** Brain tissues (hippocampus) from the two groups were homogenized and LCN2 levels were detected by immunoblotting. Actin was used as a loading control. **F** Quantitative analysis of LCN2 levels (n = 4). **G** Representative immunofluorescence images show GFAP and LCN2 fluorescence in brain slices of two groups. **H** Quantitative analysis of GFAP and LCN2 fluorescence in hippocampal slices, (n = 4). **I** Representative immunofluorescence images show GFAP and LCN2 fluorescence in primary hippocampal astrocytes. **J** Quantitative analysis of LCN2 fluorescence in primary hippocampal astrocytes (n = 9–10). **K** GFAP and LCN2 protein levels were detected in primary hippocampal astrocytes by immunoblotting, actin were used as a loading control. Quantitative analysis of the GFAP (**L**) and LCN2 (**M**), (n = 4). **N** ELISA kit was used to measure the LCN2 levels in ACM, (n = 4). Data are presented as Mean ± SEM. A non-parametric test of Mann-Whitey test was used for statistical analysis in (**F**, **H**, **L**, **M**, **N**). A two-tailed Student’s t-test was used for statistical analysis in statistical analysis in (**J**).*p < 0.05, ****p < 0.0001, versus Control group.

To uncover potential drives of sepsis/LPS-induced cognitive deficit, we used and analyzed the LPS-related dataset GSE88959 from the Gene Expression Omnibus (GEO) database. Bioinformatics analyses showed significant expression levels of differentially expressed genes (DEGs) (Fig. 1A, B). Functional enrichment analysis was conducted using GO (sFig. 2A), Reactome (sFig. 2B), and KEGG pathways (sFig. 2C) assessment. 58 genes were identified upon intersecting the DEGs with core enrichment genes in the “logFC > 2” and “immune system” (Fig. 1C). Moreover, the hub gene, LCN2 was identified through the Protein-Protein Interaction (PPI) network analysis with connectivity degree among 58 intersected genes (Fig. 1D). In fact, LCN2 is expressed at low levels in the brain, but is significantly upregulated in acute and chronic pathological responses [31]. The hippocampus, a crucial brain region involved in memory and spatial navigation, is vital for synaptic plasticity, which serves as the cellular foundation of learning and memory [32]. Our results from western blotting also revealed markedly higher levels of LCN2 protein level of hippocampus in the LPS group compared to controls (Fig. 1E, F). In fact, we also measured the LCN2 levels in the hippocampus at 8 h, 24 h, 7 days, and 14 days after intraperitoneal injection of LPS, and found that LCN2 levels were increased in the hippocampus after intraperitoneal injection of LPS 1 week and decreased after 14 days (sFig. 3A, B). This indicates that an increased LCN2 may induce cognitive impairments. In the central nervous system, reactive astrocytes undergo morphological and functional remodeling during brain injury and infection to regulate neuroinflammation and pathological outcomes [7, 33]. Moreover, reactive astrocytes are the main source of LCN2 following brain insult, and LCN2 is considered to be a marker of early reactive astrocyte proliferation and mediates the toxicity of neurons [34–36]. The immunofluorescence experiment revealed an elevated astrocytic LCN2 fluorescence level of hippocampus following LPS exposure (Fig. 1G, H). Consistently, similar results were observed when primary hippocampal astrocytes were treated with 1 μg/ml LPS (Fig. 1I, J). In addition, this is also confirmed by western blotting tests (Fig. 1K–M), as well as ELISA test results from astrocyte-conditioned medium which revealed a significant increase in LCN2 levels in the LPS group compared to the control group (Fig. 1N). We also detected the levels of LCN2 in microglia. The immunofluorescence experiment revealed an elevated microglia LCN2 in the hippocampus of the LPS mice (sFig. 4A, B). which is consistent with the Jin et al. study [15]. Collectively, these findings suggest that LPS may contribute to sepsis-related cognitive impairments through the significant upregulation of glia-released LCN2.

### LCN2 induced synaptic damage and cognitive deficits

Upregulated LCN2 is associated with a variety of central nervous system diseases, such as acute brain injury ischemic stroke, hemorrhagic stroke, traumatic brain injury, and neurodegenerative brain diseases such as Alzheimer’s disease (AD), Parkinson’s disease (PD), and vascular dementia (VaD) [36–39]. Bioinformatics analyses of LCN2-treated primary hippocampal neurons obtained from NCBI [17] showed significant expression levels of differentially expressed genes (DEGs) (sFig. 5A). Functional enrichment analysis was conducted using KEGG (sFig. 5B) pathway assessment, further supporting that increased LCN2 might be associated with metabolic dysfunction and CNS diseases.

**Fig. 2:**
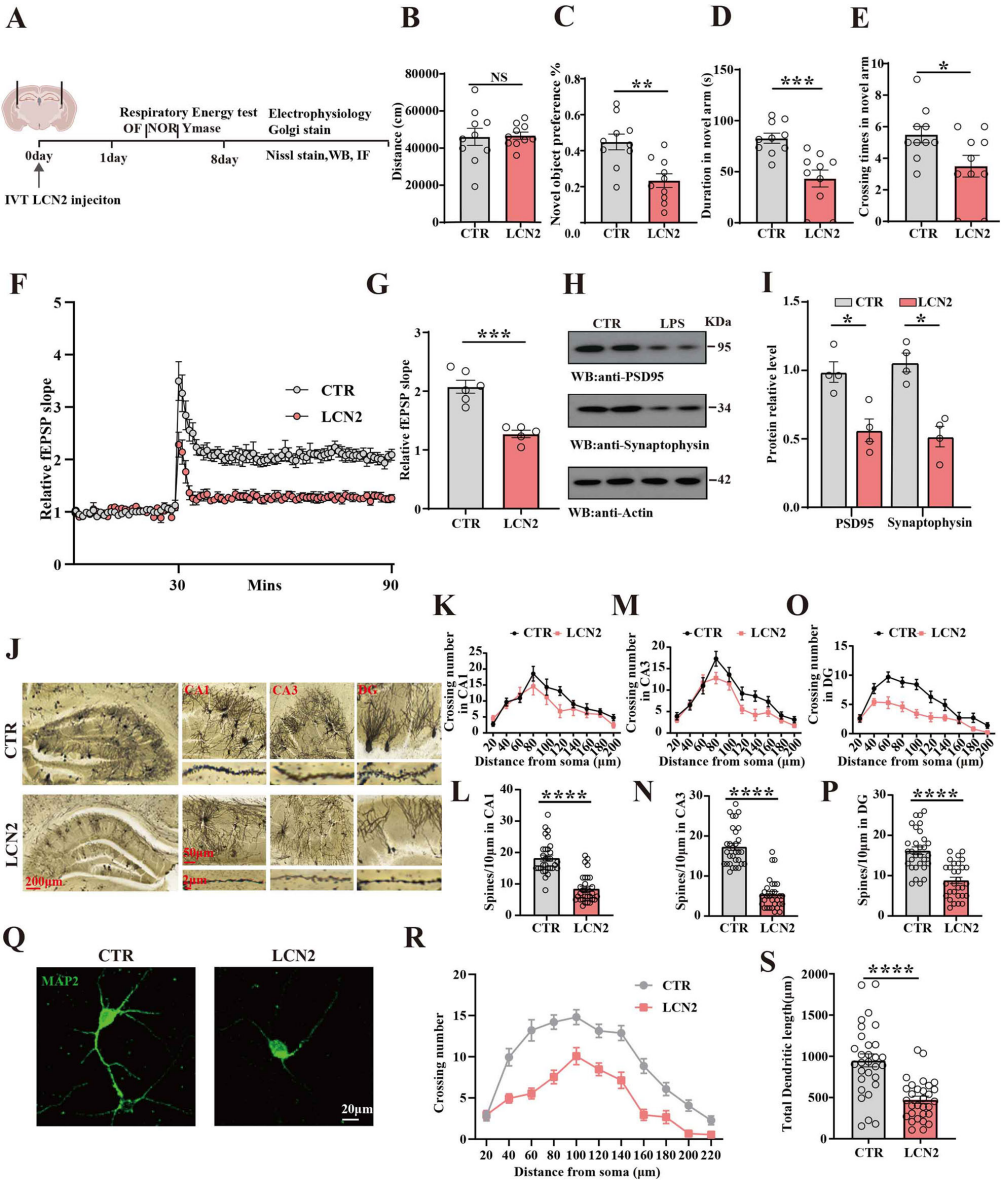
LCN2 induced synaptic damage and cognitive deficits. **A** Experimental design sketch. C57 mice were randomly divided into 2 groups. In the control group, 10 mice were injected intraperitoneally with PBS. In the model (Mod) group, 10 mice were bilaterally injected with LCN2 in the lateral ventricles. Following the treatment, behavioral, electrophysiological, and biochemical tests were performed. **B** The open-field test measured the total distance covered in the two groups, (n = 10). **C** The novel object recognition test measured the novel object preference, (n = 10). Y-mase test: the duration (**D**) and crossing number (**E**) in the novel arm were measured. **F** Hippocampal DG-CA1 Long-Term Potentiation was recorded using the MED64 system. **G** The normalized mean slope of fEPSPs was recorded in hippocampal slices, (n = 5–6 slices from 5 to 6 mice). **H** Brain tissues (hippocampus) from the two groups were homogenized and synaptic associated protein were detected by immunoblotting. Actin was used as a loading control. **I** Quantitative analysis of the PSD95, SYT, (n = 4). **J** Representative dendrite from Golgi-impregnated hippocampal neurons, (Scale bar: 200 μm). Sholl analysis of CA1 region (**K**), CA3 region (**M**), DG region (**O**) (Scale bar: 50 μm), (n = 10), and averaged spine density of CA1 region (**L**), CA3 region (**N**), DG region (**P**), (Scale bar: 2 μm), (n = 30). **Q** Primary hippocampal neurons were treated with LCN2, and the MAP2 was measured by immunofluorescence. Sholl analysis (**R**) (Scale bar: 20 μm), (n = 15) and quantitative analyses of dendritic length (**S**), (n = 30 hippocampal neurons) were performed. Data are presented as Mean ± SEM. A two-tailed Student’s t-test was used for statistical analysis, a non-parametric test of Mann-Whitey test was used for statistical analysis in (**I**). *p < 0.05, **p < 0.01, ***p < 0.001, ****p < 0.0001, versus Control group.

Here, to further evaluate the effect of LCN2 on cognitive function, C57 mice were randomly divided into LCN2 and control groups. Behavioral, electrophysiological, and biochemical tests were performed at 24 h after LCN2 injection (Fig. 2A). Open-field test data indicated no significant difference in the total distance covered (Fig. 2B). NOR results revealed a reduced preference in exploring new objects in the LCN2 group compared to the control group (Fig. 2C). Y-maze test showed that LCN2 mice exhibit a significantly decreased duration (Fig. 2D) and crossing times (Fig. 2E) in the novel arm compared to the control group. The electrophysiology experiments demonstrated that LCN2 reduced the slope of field excitatory postsynaptic potential (fEPSP) after high-frequency stimulation (HFS) in LCN2 mice compared to the control (Fig. 2F, G). Consistently, western blotting data revealed significant decrease in postsynaptic density protein95 (PSD95) and synaptophysin (SYT) in the LCN2 group compared to the control group (Fig. 2H, I). Using Golgi staining (Fig. 2J), we further analyzed the dendritic architecture of hippocampal neurons of CA1 (Fig. 2K), CA3 (Fig. 2M), and DG region (Fig. 2O). This revealed a marked decrease in the dendritic complexity in the LCN2 group compared to the control group. Moreover, a significant reduction in the dendritic spine density (Fig. 2L, N, P) was also observed. Additionally, primary hippocampal neurons were treated with the recombinant LCN2 protein 100 ng/ml, resulting in an obvious decrease in the dendritic complexity (Fig. 2Q, R) and the total dendritic length (Fig. 2S) as depicted by immunofluorescence staining with anti-MAP2 antibody. These findings collectively indicate that LCN2 may contribute to synaptic and cognitive impairments.

### Downregulation of LCN2 alleviated LPS-induced neuronal damage

**Fig. 3:**
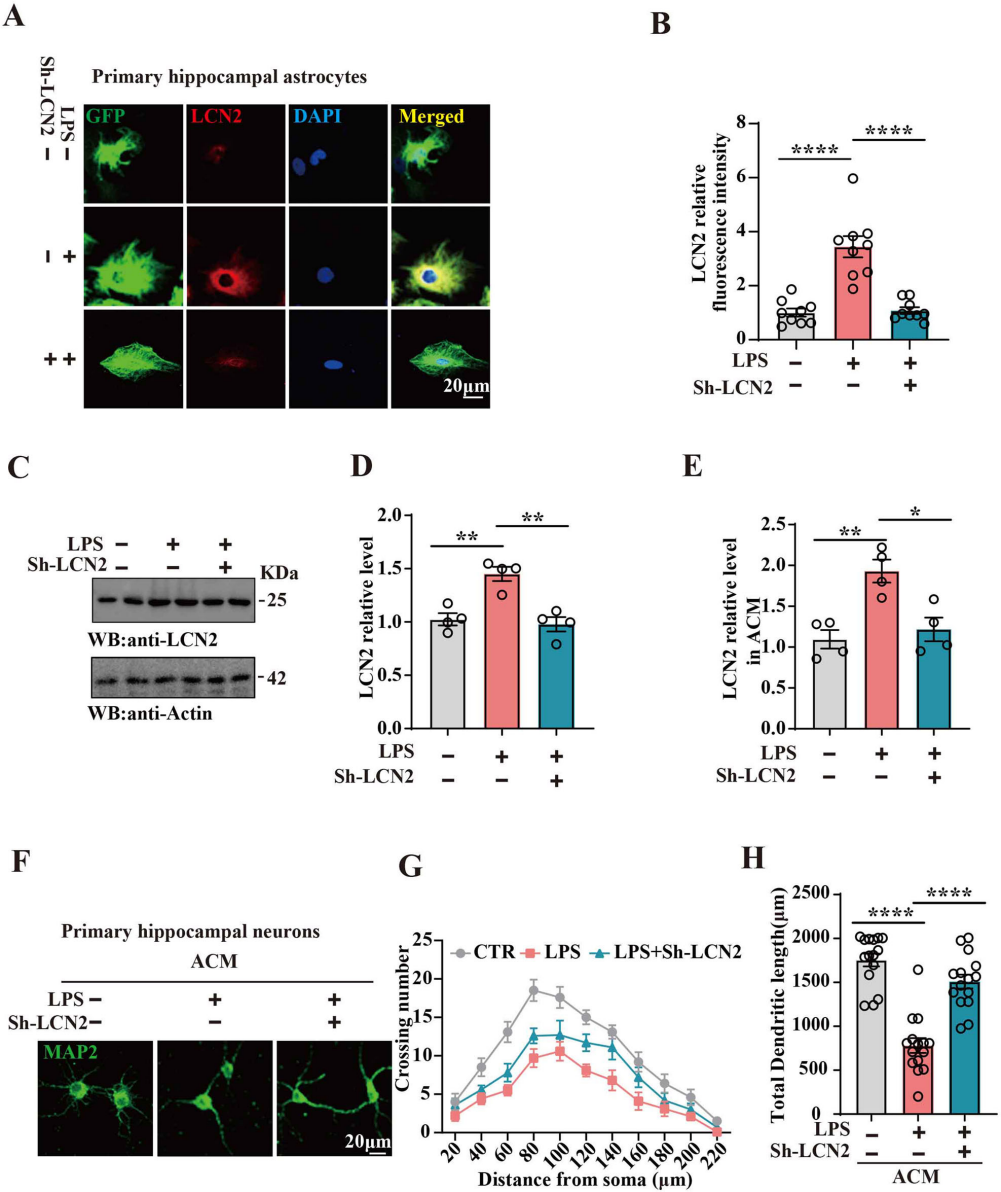
Downregulation of LCN2 alleviated LPS-related neuronal damage. **A** Representative immunofluorescence images show astrocyte activation and LCN2 fluorescence in primary hippocampal astrocytes. **B** Quantitative analysis of LCN2 fluorescence in primary hippocampal astrocytes (n = 9–10). **C** LCN2 protein levels were detected in primary hippocampal astrocytes by immunoblotting, actin was used as a loading control, and **D** quantitative analysis of the LCN2, (n = 4). **E** ELISA kit was used to measure the LCN2 levels in ACM, (n = 4). **F** Primary hippocampal neurons were treated with Control-ACM, LPS-ACM, AAV-Sh-LCN2-ACM, and the MAP2 was measured by immunofluorescence. Sholl analysis (Scale bar: 20 μm), (n = 10) (**G**) and quantitative analyses of dendritic length (n = 15) (**H**) were performed. Data are presented as Mean ± SEM. one-way ANOVA was used for statistical analysis. *p < 0.05, **p < 0.01, ****p < 0.0001, versus LPS group.

As mentioned above, reactive astrocytes are the main source of LCN2 after neuroinflammation [36]. To explore the role of LCN2 in LPS-induced neuronal damage, primary hippocampal astrocytes were treated with 1 μg/ml LPS and AAV-Sh-LCN2. Immunofluorescence revealed that AAV-Sh-LCN2 reduced astrocytic LCN2 fluorescence levels (Fig. 3A, B). Western blotting results (Fig. 3C, D) as well as ELISA test results from astrocyte-conditioned medium (ACM) (Fig. 3E) showed a decrease in LCN2 protein levels in the LPS+Sh-LCN2 group compared to the LPS group. To explore whether astrocyte-derived LCN2 causes neuronal damage, we culture primary hippocampal neurons using ACM (Fig. 3F) and found LPS-ACM induced anomalies in morphology of neurons (Fig. 3F–H). Interestingly, LPS+Sh-LCN2-ACM treated neurons exhibited a significantly higher dendritic complexity and increased total dendritic length compared to the LPS-ACM-treated ones, as depicted by immunofluorescence staining with anti-MAP2 antibody (Fig. 3F–H). These findings suggest that astrocyte-released LCN2 induced LPS-related neuronal damage.

### Downregulation of LCN2 alleviated sepsis-related synaptic and cognitive impairments

**Fig. 4:**
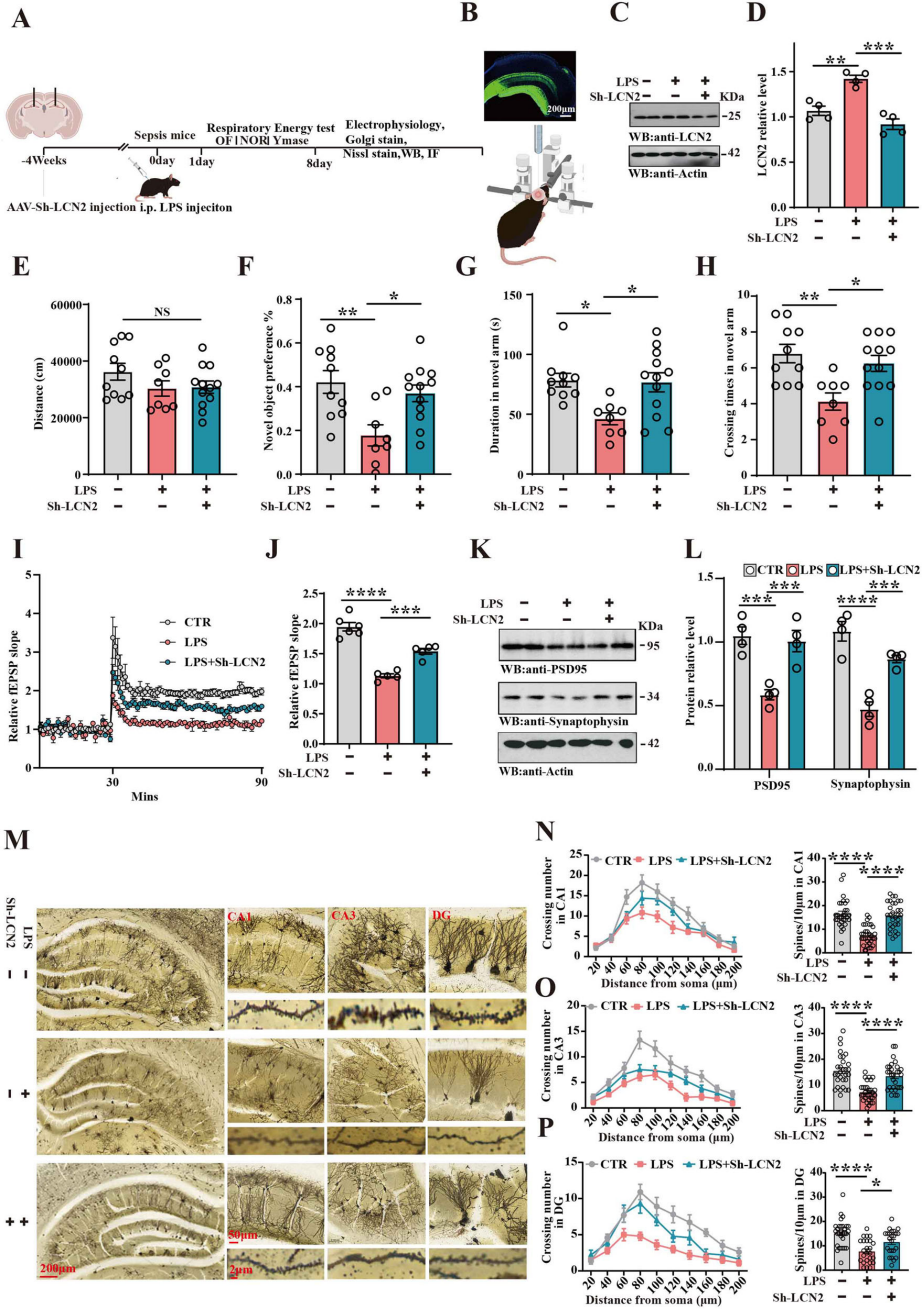
Downregulation of LCN2 alleviated sepsis-related synaptic and cognitive impairments. **A** Experimental design sketch. C57 mice were randomly divided into 3 groups. In the control group, mice were bilaterally injected with AAV-Sh-vector in the DG region and injected intraperitoneally with normal saline. In the model (Mod) group, mice were bilaterally injected with AAV-Sh-vector in the DG region and injected intraperitoneally with LPS. In the preventive (Pre) group, mice were bilaterally injected with AAV-Sh-LCN2 in the DG region and received intraperitoneal injections of LPS. Following the treatment, behavioral, electrophysiological, and biochemical tests were performed. **B** Fluorescence microscopy confirmed GFP expression in the hippocampus 4 weeks after injection. **C** Brain tissues (hippocampus) were homogenized, and LCN2 protein level was detected by immunoblotting. **D** Quantitative analysis of LCN2 expression was conducted, (n = 4). **E** The open-field test measured the total distance covered in the three groups, (n = 8–12). **F** The novel object recognition test measured the preference, (n = 8–12). Y-mase test: the duration **G** and crossing number **H** in the novel arm were measured, (n = 8–12). **I** Hippocampal DG-CA1 Long-Term Potentiation was recorded using the MED64 system. **J** The normalized mean slope of fEPSPs was recorded in hippocampal slices, (n = 5–6 slices from 5 to 6 mice). **K** Brain tissues (hippocampus) from the three groups were homogenized and synaptic associated protein were detected by immunoblotting. Actin was used as a loading control. **L** Quantitative analysis of the PSD95, SYT, (n = 4). **M** Representative dendrite from Golgi-impregnated hippocampal neurons, (Scale bar: 200 μm). Sholl analysis (Scale bar: 50 μm), (n = 10) and averaged spine density of CA1 region, (Scale bar: 2 μm), (n = 30) (**N**). Sholl analysis (Scale bar: 50 μm), (n = 10), and averaged spine density of CA3 region, (Scale bar: 2 μm), (n = 30) (**O**). Sholl analysis (Scale bar: 50 μm), (n = 10) and averaged spine density of DG region (Scale bar: 2 μm), (n = 30) (**P**). Data are presented as Mean ± SEM, one-way or two-way ANOVA was used for statistical analysis. *p < 0.05, **p < 0.01, ***p < 0.001, ****p < 0.0001, versus LPS group.

To investigate whether LCN2 plays an important role in sepsis-related cognitive impairments, C57 mice were randomly allocated into three groups (control, LPS, and LPS+Sh-LCN2). Behavioral, electrophysiological, and biochemical tests were performed (Fig. 4A). After 4 weeks AAV infection, immunofluorescence showed the GFP as a report protein observed in the hippocampal region (Fig. 4B) and a decreased LCN2 levels in the LPS+Sh-LCN2 group (Fig. 4C, D). Open-field test data showed no significant difference in the total distance covered (Fig. 4E). However, the NOR test showed an increased preference for exploring new things as indicated (Fig. 4F) in the LPS+ Sh-LCN2 group compared to the Mod group. In support of this, the data from the Y-maze test revealed that downregulation of LCN2 led to a significantly increased duration (Fig. 4G) and crossing number in the novel arm (Fig. 4H). The electrophysiology experiments data showed that downregulation of LCN2 improved the slope of field excitatory postsynaptic potential after high-frequency stimulation compared to the Mod group (Fig. 4I, J). Consistently, western blotting data revealed a significant increase in PSD95 and SYT levels in the LPS+Sh-LCN2 group compared to the Mod group (Fig. 4K, L). In addition, we further examined the dendrite of hippocampal neurons. Golgi staining (Fig. 4M) showed that downregulation of LCN2 resulted in an obvious increase in dendritic complexity, as well as dendritic spine density in the CA1, CA3, and DG region (Fig. 4N–P). These findings collectively indicate that the downregulation of LCN2 may alleviate sepsis-induced synaptic, learning, and memory impairments. We also investigated whether synaptic and memory deficits persist over a relatively longer period. We found that the downregulation of LCN2 alleviated sepsis-related cognitive impairments (sFig. 6B–E) and synaptic dysfunction (sFig. 6F–H) 2 weeks after intraperitoneal injection of LPS (sFig. 6A). We thought that an increase in LCN2 level at an early stage induced lasting damage.

### Downregulation of LCN2 improved Sepsis-induced neuronal loss

**Fig. 5:**
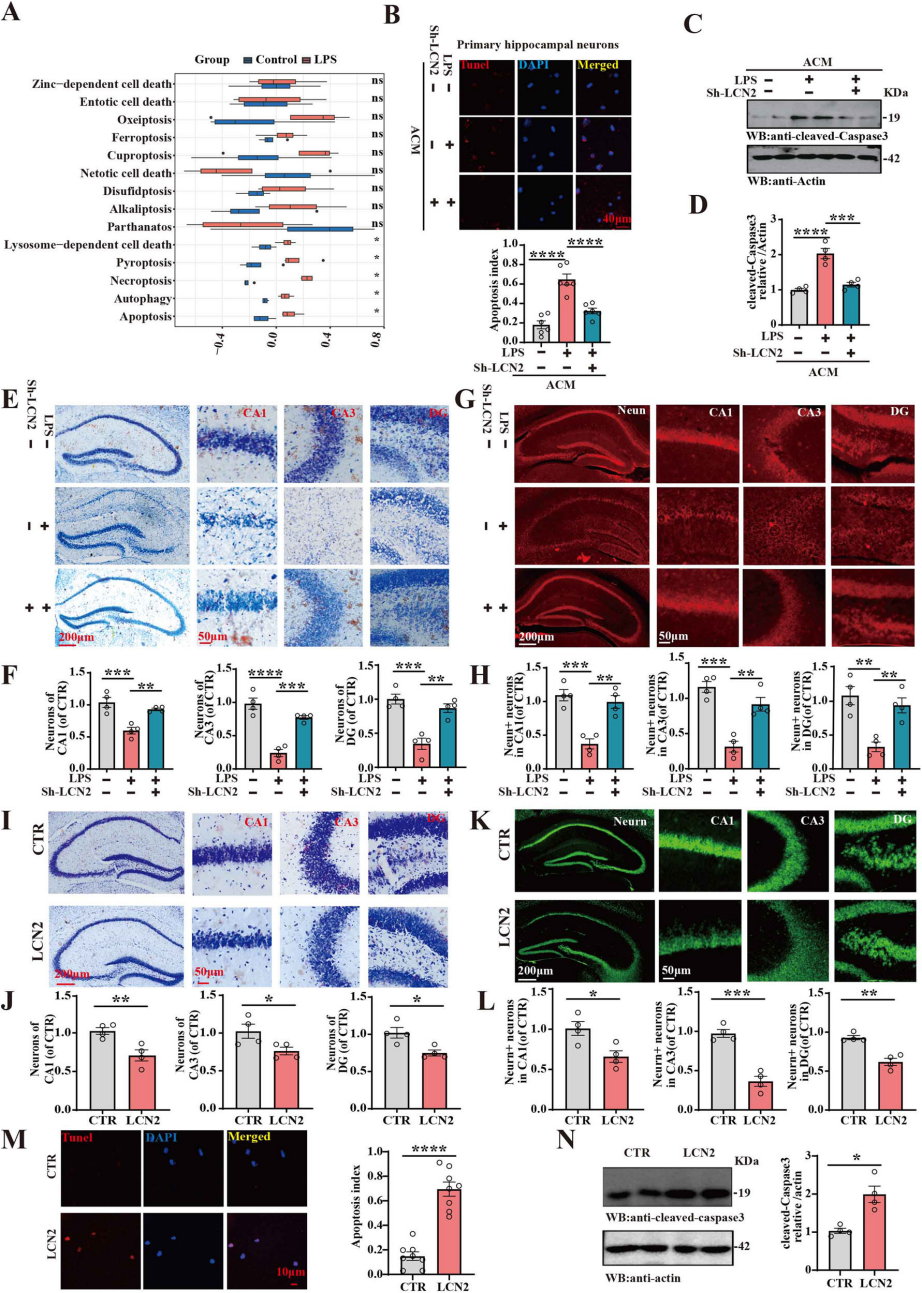
Downregulation of LCN2 improved sepsis-induced neuronal loss. **A** Enrichment score of different cell death in dataset GSE88959. **B** Representative images of TUNEL staining of primary hippocampal neurons treated with Control-ACM, LPS-ACM, AAV-Sh-LCN2-ACM, quantitative analysis of apoptosis index in primary hippocampal neuron (n = 6–8). **C** cleaved caspase-3 was detected by immunoblotting in primary hippocampal neurons. Actin was used as a loading control. **D** Quantitative analysis of the cleaved caspase-3, (n = 4). **E** Representative images of Nissl’s staining of brain slices. **F** Quantitative analysis of cell numbers of CA1, CA3, DG region (n = 4). **G** Representative images of Neun staining of brain slices. **H** Quantitative analysis of Neun+ number of CA1, CA3, DG region (n = 4). **I** Representative images of Nissl’s staining. **J** Quantitative analysis of cell number of CA1, CA3, DG region (n = 4). **K** Representative images of Neun staining. **L** Quantitative analysis of Neun staining number of CA1, CA3, DG region (n = 4). **M** Representative images of TUNEL staining of primary hippocampal neurons treated with control or LCN2, quantitative analysis of apoptosis index in primary hippocampal neurons (n = 8). **N** cleaved caspase-3 was detected by immunoblotting in primary hippocampal neurons. Actin was used as a loading control (n = 4). Data are presented as Mean ± SEM. A two-tailed Student’s t-test was used to assess the variance between two groups, a non-parametric test of Mann-Whitey test was used for statistical analysis in (**N**). One- or two-way ANOVA was used for the three groups. *p < 0.05, **p < 0.01, ***p < 0.001, ****p < 0.0001, versus LPS or Control group.

As a key factor of SAE, inflammation is associated with neuronal death [40–42]. Meanwhile, it has been reported that LCN2 has many biological functions in mediating innate immune response, inflammatory response, iron homeostasis, cell migration and differentiation, energy metabolism, and cell death [35]. To explore the molecular mechanisms of sepsis-mediated neuronal loss, we firstly made cell death enrichment analysis and found that the LPS group exhibited neuronal cell death pathways (Fig. 5A). Next, we explored the effects and underlying mechanisms of LCN2 on sepsis-related neuronal death using primary hippocampal neurons treated with ACM. Interestingly, the downregulation of LCN2 in astrocyte rescued neuronal apoptosis caused by LPS + ACM as suggested by TUNEL staining (Fig. 5B) and we noted the elevated levels of cleaved caspase-3, indicating heightened apoptosis in these LPS-induced brain regions. Importantly, the downregulation of LCN2 effectively abrogated these effects (Fig. 5C, D). Nissl’s staining (Fig. 5E, F) and immunofluorescence with NeuN (Fig. 5G, H) unveiled that downregulation of LCN2 alone is efficient enough to reverse the reduction in neuronal population caused by LPS in the hippocampus of mice. Moreover, LCN2 injection induced a significant reduction of hippocampal neurons compared to the control group (Fig. 5I–L). In addition, TUNEL staining and western blotting also demonstrated that LCN2 induced apoptosis in primary hippocampal neurons (Fig. 5M, N). Together, these findings strongly support that LCN2 might play a significant role in the neuronal loss associated with LPS-related synaptic and cognitive impairments.

### Downregulation of LCN2 resisted oxidative stress and mitochondrial dysfunction caused by LPS

**Fig. 6:**
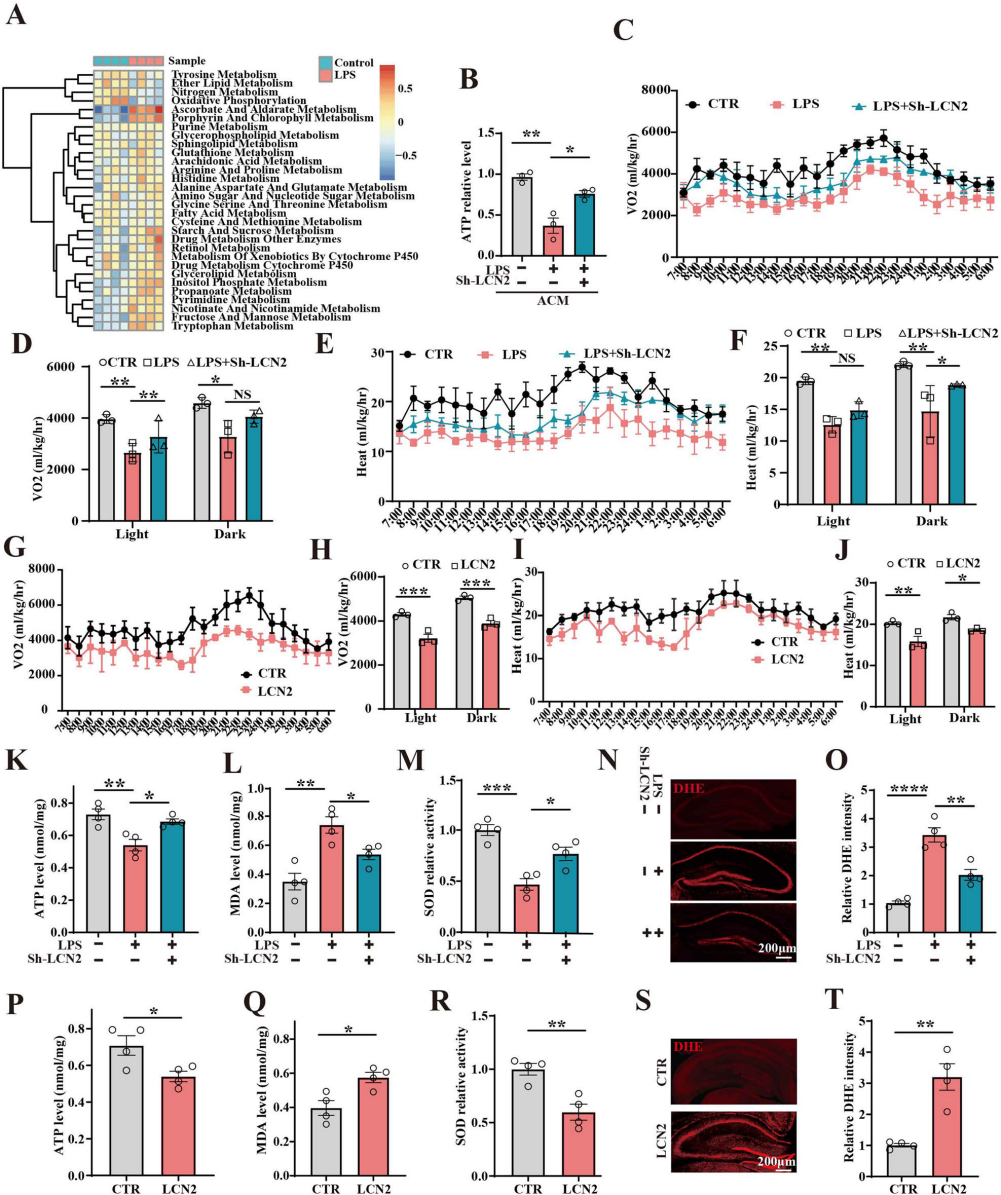
Downregulation of LCN2 resisted oxidative stress. **A** Heatmap illustrating different metabolism pathways enrichment results in dataset GSE88959. **B** The ATP levels were tested in primary hippocampal neurons (n = 3). **C** VO2 was tested in 24 h of three groups. **D** Quantitative analysis the VO2 of Light and Dark (n = 3). **E** the heat was tested in 24 h. **F** Quantitative analysis of Light and Dark (n = 3). **G** VO2 was tested in 24 h of control and LCN2 groups. **H** Quantitative analysis of Light and Dark (n = 3). **I** the heat was tested in 24 h. **J** Quantitative analysis of Light and Dark (n = 3). **K** The ATP production was tested in the hippocampal of three groups (n = 4). **L** The MDA levels were tested in the hippocampal of three groups (n = 4). **M** The total SOD activity was tested in the hippocampal of three groups (n = 4). **N**, **O** ROS production was detected by DHE dye in the hippocampal of three groups. The representative DHE fluorescence images (**N**) and quantitation (n = 4) (**O**). **P** The ATP production was tested in the hippocampal of LCN2 and control groups (n = 4). **Q** The MDA levels were tested in the hippocampal of LCN2 and control group (n = 4). **R** The total SOD activity was tested in the hippocampal of LCN2 and control group (n = 4). The representative DHE fluorescence images (**S**) and quantitation (**T**) of LCN2 and control group (n = 4). Data are presented as Mean ± SEM. A two-tailed Student’s t-test was used to assess the variance between two groups. One- or two-way ANOVA was used for the three groups. *p < 0.05, **p < 0.01, ***p < 0.001, ****p < 0.0001, versus LPS or Control group.

Accumulating data showed that inflammation triggers the activation of glia, which further release cytokines and reactive oxygen species, leading to oxidative stress and neuronal damage [43]. Studies have shown that oxidative stress mainly mediates apoptosis through mitochondrial and endoplasmic reticulum stress [44, 45]. To explore the molecular mechanisms of sepsis-mediated neuronal loss, different metabolism pathways enrichment was shown in dataset GSE88959, Heatmap illustrating showed that the LPS group downregulated the oxidative phosphorylation pathway (Fig. 6A). It was also observed that LPS+Sh-LCN2-ACM rescued the LPS-induced reduction in ATP level in primary hippocampal neurons (Fig. 6B). In addition, mice respiratory energy and general behavior monitoring data showed a significantly increased VO_2_ in the LPS+Sh-LCN2 group compared to the LPS group (Fig. 6C, D), as well as a significant increase in heat production (Fig. 6E, F). Consistently, a significant reduction in VO_2_ (Fig. 6G, H) and heat production (Fig. 6I, J) was observed in the LCN2 group compared to the control group.

Studies have shown that in addition to supplying energy to cells, mitochondria also participate in processes such as cell differentiation, cell information transmission, and apoptosis, and have the ability to regulate cell growth and cell cycle, including ATP synthesis, ROS production, and clearance, and the complex regulation of apoptosis throughout the cell life cycle [46–48]. More and more studies have highlighted the relationship between mitochondrial dysfunction and neuronal apoptotic in SAE pathophysiology [49, 50]. Our data showed that downregulation of LCN2 rescued the LPS-induced reduction of ATP levels (Fig. 6K), elevation in malondialdehyde (MDA) (Fig. 6L), decrease in superoxide dismutase (SOD) activity (Fig. 6M), and accumulation of ROS, as assessed by dihydroethidium (DHE) staining (Fig. 6N, O) in the hippocampus of mice. Moreover, the LCN2 group showed reduced ATP levels (Fig. 6P), elevated MDA (Fig. 6Q), decreased SOD activity (Fig. 6R), and accumulated ROS (Fig. 6S, T). These results indicated that LPS-induced SAE in mice may be due to LCN2-mediated mitochondrial impairment.

**Fig. 7:**
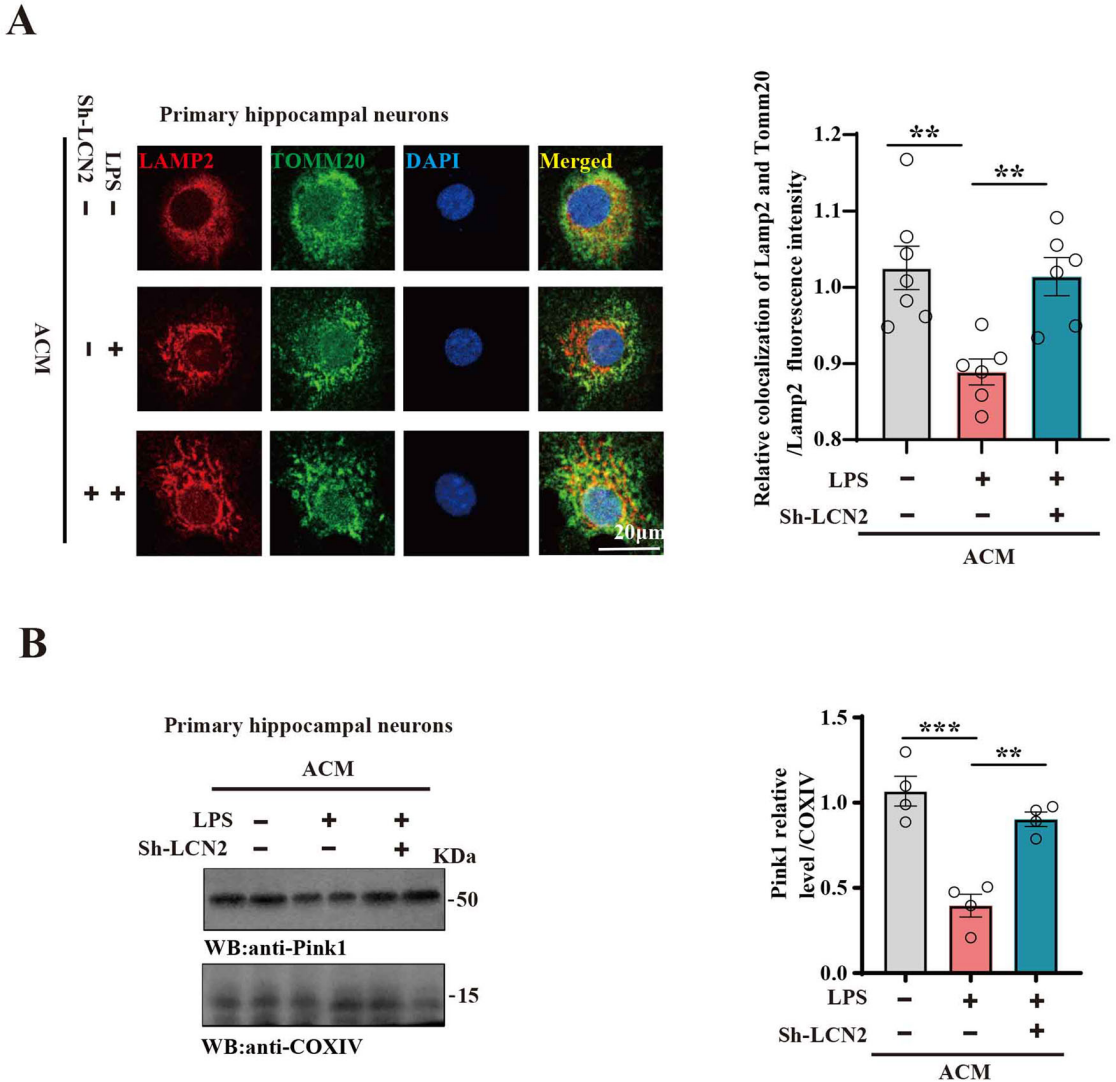
Downregulation of LCN2 resisted mitochondrial dysfunction. **A** Primary hippocampal neurons were treated with ACM. colocalization of Lamp2 (lysosome marker) and Tomm20 (mitochondrial marker) by immunofluorescence. Quantitative analysis of colocalization of Lamp2 and Tomm20/Lamp2 fluorescence in primary hippocampal neurons (n = 6–7). **B** The mitochondrial portions were isolated, and Pink1 protein levels were detected by immunoblotting in primary hippocampal neurons, COX IV was used as a loading control. Quantitative analysis of Pink1 expression was conducted (n = 4). Data are presented as Mean ± SEM. One-way ANOVA was used for the three groups. **p < 0.01, ***p < 0.001, versus LPS group.

The above results suggest that downregulation of LCN2 may have the ability of mitochondrial protection. Next, we cultured primary hippocampal neurons by ACM. Interestingly, results from immunofluorescence showed that LPS-ACM reduced colocalization of Lamp2 (lysosome marker) and Tomm20 (mitochondrial marker) when compared with control-ACM treated neurons, while LPS+Sh-LCN2-ACM restored the colocalization (Fig. 7A). Additionally, we isolated the mitochondrial portion of primary hippocampal neurons and found that Pink1 (autophagy marker) was reduced after incubation with LPS-ACM. Notably, the downregulation of LCN2 reversed the level of Pink1 (Fig. 7B). These findings further supported that mitochondrial impairment is involved in SAE, and downregulation of LCN2 may rescue SAE through restoration of mitochondrial function.

## Discussion

Inflammation is an integral pathological process of several nervous system diseases, including sepsis-associated encephalopathy (SAE) [51]. In the current study, we elucidated an abnormal upregulation of LCN2 protein in the LPS-induced mouse model of sepsis, which correlates with neuronal loss in the hippocampus. Our findings revealed that neuronal loss in sepsis mice results from LCN2 upregulation. The increased LCN2 leads to mitochondrial dysfunction and oxidative stress. Downregulation of LCN2 alleviated neuronal damage and significantly improved synaptic and cognitive impairments in sepsis mice. Thus, our findings suggest a previously undiscovered etiopathogenic relationship between sepsis and cognitive impairments that is linked to an increased LCN2 and the potential of LCN2-based therapeutics for encephalopathy such as SAE.

Currently, cecal ligation and puncture (CLP) and LPS treatment are the most widely used classical methods to replicate sepsis models [52–57]. Compared to LPS treatment, CLP causes an invasive trauma with a higher mortality rate, leading to a relative challenge to perform behavioral tests on mice 24 h after surgery. Therefore, we used LPS-induced sepsis in the present study. We here showed that LCN2 may play a key role triggering the occurrence of SAE. LCN2 has many biological functions in mediating innate immune response, inflammatory response, iron homeostasis, cell migration and differentiation, energy metabolism, and cell death [10, 35]. We also measured the LCN2 levels in the hippocampus at 8 h, 24 h, 7 days, and 14 days after intraperitoneal injection of LPS, and found that LCN2 levels were decreased after 14 days (sFig. 3). We thought that an increase in LCN2 levels at an early stage induced lasting damage. An increased LCN2 at early sepsis may act as a potential biomarker for sepsis-associated encephalopathy. Moreover, our bioinformatics analysis identified LCN2 as one of the hub genes in infection and neurological inflammation. Bilateral injection of recombinant LCN2 protein into the lateral ventricles induced hippocampal neuronal loss, synaptic and cognitive impairments, suggesting that LCN2 is implicated in the pathogenesis of SAE. These findings strongly indicate a critical role for LCN2 in the pathological and behavioral alterations observed in sepsis-related cognitive impairments.

Our findings indicate that sepsis reduces metabolism and causes mitochondrial damage. Mitochondrial damage, in turn, can exert adverse biological effects on tissues and cells by activating intracellular signal transduction pathways, leading to the upregulation of cytokines and ROS (oxidative stress) [58, 59]. Oxidative stress contributes to various events that result in cell damage, including increased membrane rigidity, DNA strand breaks, and impaired glucose uptake. Furthermore, antioxidant depletion or reduced energy production due to disturbed glucose metabolism diminishes the cell’s capacity to counteract radical-induced damage to membranes, proteins, and DNA [60]. These observations suggest a potential mechanism by which sepsis dysregulates lipid or glucose metabolism by upregulating LCN2 levels, thereby affecting mitochondrial function, causing mitochondrial damage, and ultimately leading to neuronal death, which is worthy of further investigation.

LCN2 is a pleiotropic molecule that is induced in central nervous system diseases. Some studies showed that LCN2 plays a protective role by sequestering iron from pathogens, thereby combating bacterial infections to some extent. However, other studies indicate that LCN2 exhibits neurotoxic effects [17, 35, 61]. In our study, we observed a significant increase in LCN2 levels, resulting in mitochondrial dysfunction, oxidative stress and neuronal damage.

## Limitation

In the present study, we identified the role of LCN2 secreted by astrocytes in SAE, we propose that knocking out LCN2 in astrocytes could be a more effective approach. Besides, we have detected the levels of LCN2 in microglia and found an elevated microglia LCN2 in the hippocampus of the LPS mice (sFig. 4), which is consistent with the Jin et al. study [15]. So, future studies will investigate the effects of microglia-released LCN2 on neurons. Moreover, our study demonstrated that astrocyte-secreted LCN2 damages neurons, although we did not specifically target its receptor. Current evidence suggests that LCN2 primarily exerts its effects via the 24p3R receptor. In upcoming studies, we plan to target the 24p3R receptor to elucidate the mechanisms through which LCN2 causes neuronal damage. Lastly, further research is necessary to determine how LCN2 damages neuronal mitochondria and induces oxidative stress.

## Conclusion

**Fig. 8:**
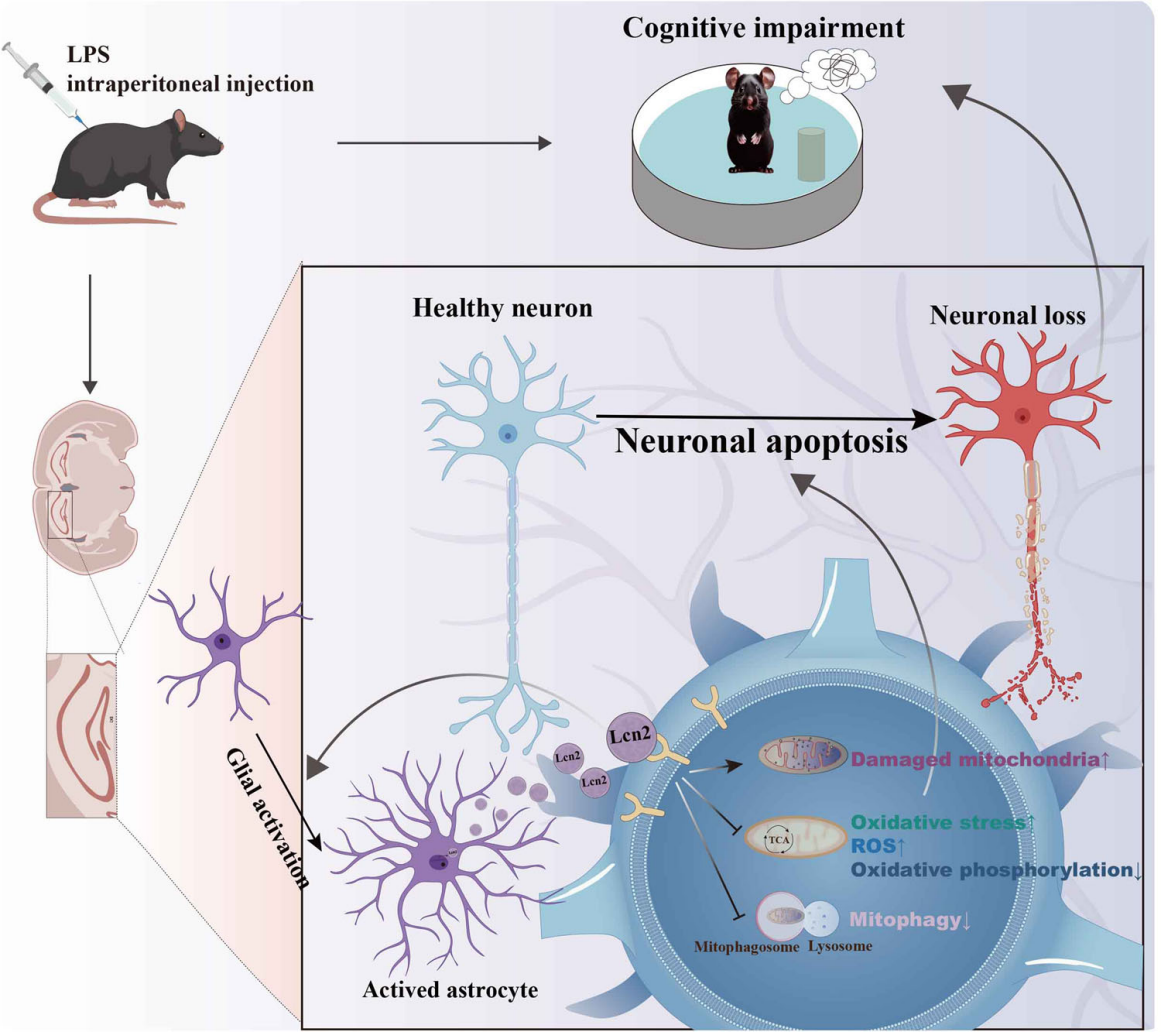
Schematic illustration of the effects of LCN2 in the sepsis brain. Sepsis upregulates LCN2 triggering oxidative stress, mitochondrial dysfunction, and neuronal loss, thus leading to synaptic damage and cognitive impairments.

In summary, our study provides a novel pathogenic link between sepsis and encephalopathy, in which sepsis upregulates LCN2, triggering mitochondrial dysfunction and neuronal loss, thus leading to synaptic and cognitive impairments (Fig. 8). Given the deteriorative effects of LCN2 in SAE, downregulation of LCN2 might be a potential strategy for the treatment of sepsis-associated cognitive dysfunction.

## Material and methods

### Animals

Eight-week-old male C57 mice were obtained from the Shulaibao Company. The animals were assigned to different experimental groups based on the litter in a way that every experimental group had similar number of males. The mice were housed under standard laboratory conditions with a 12-h light-dark cycle. According to the research requirements, the C57 mice were randomly assigned to various experimental groups. All animal procedures were conducted in accordance with the guidelines of the Institutional Animal Care and Use Committee of Tongji Medical College, Huazhong University of Science and Technology, ensuring strict adherence to ethical standards.

Inhere, LPS-induced sepsis mice model was used. Briefly, 8-week-old male C57 mice were randomly divided into two groups. The model and control groups were intraperitoneally injected with LPS (10 mg/kg) or normal saline, respectively. To explore the effect of LCN2 on cognitive function, 8-week-old C57 mice were randomly divided into two groups (LCN2 and control). We performed bilateral intraventricular injections of recombinant LCN2 protein (50 μg/kg), with PBS serving as the control. In addition, to investigate the role of LCN2 in sepsis-related cognitive impairments, mice in the control group were given AAV-vector injections into the DG region and intraperitoneal injections with PBS. AAV-vector injection into the DG region and intraperitoneal injection with LPS (10 mg/kg) were performed to establish the Mod group. The third group of mice received AAV-Sh-LCN2 injections into the DG region and intraperitoneal injections of LPS, serving as the LPS+Sh-LCN2 group.

### Stereotactic surgery

The C57 mice were anesthetized using isoflurane and positioned in a stereotactic apparatus. After disinfecting with iodophor and 75% alcohol, the scalp was incised along the midline between the ears. Stereotactic holes were drilled at the following coordinates relative to bregma: posterior 0.7 mm, lateral 1.2 mm, and depth 2.2 mm. The World Precision Instruments microinjection system was used to perform bilateral injections of recombinant LCN2 protein into the lateral ventricles at a rate of 0.125 μL/min. In addition, AAV2/9-CAG-MasterRNAi155e(Lcn2)-eGFP-WPRE-pA was injected into the DG region. Stereotactic holes were drilled at the following coordinates relative to bregma: posterior 2.18 mm, lateral 1.2 mm, and depth 2.2 mm. After a 10-min infusion, the needle was slowly removed, and the incision was sutured.

### Reagents

LPS was obtained from Sigma Company (Product NO. L2880 and L4391). Recombinant LCN2 protein was procured from MCE Company (Product No. HY-P70658A). The antibodies used in this study are listed in Table 1.

**Table 1:** Primary antibodies used in this study.

Antibodies	Types	Specificity	Dilution	Source
Beta actin	mAb	Beta actin	1:2000 for WB	Proteintech (66009-1-Ig)
LCN2	pAb	Lipocalin-2/NGAL	1:1000 for WB 1:300 for IF	R&D Systems (AF1857)
GFAP	mAb	GFAP (GA5)	1:1000 for WB 1:500 for IF	Cell signaling (3670)
Cleaved Caspase-3	pAb	Cleaved Caspase-3 (Asp175)	1:1000 for WB	Cell signaling (9661S)
COX IV	pAb	COX IV	1:1000 for WB	Abclonal (A6564)
Pink1	mAb	PINK1 (38CT20.8.5)	1:1000 for WB	Santa Cruz (SC-517353)
NeuN	mAb	NeuN (D4G4O)	1:500 for IF	Cell Signaling (24307)
TOMM20	mAb	TOMM20 (EPR15581-39)	1:300 for IF	Abcam(ab186734)
LAMP2	mAb	LAMP2 (H4B4)	1:300 for IF	Santa Cruz(SC-18822)
PSD95	mAb	PSD95 (7E3)	1:1000 for WB	Cell Signaling (36233)
Synaptophysin	pAb	Synaptophysin	1:1000 for WB	Proteintech (17785-1-AP)
MAP2	mAb	MAP2	1:300 for IF	Sigma (M9942)

*mAb* monoclonal Antibody, *pAb* polyclonal Antibody, *WB* Western blotting, *IF* Immunofluorescence.

### Behavior tests

#### Open-field test

The test apparatus consists of a standard open field: a 50 × 50 × 50 cm open space. The floor of the arena was divided into nine equal sections. C57 mice were individually allowed to explore the arena for 5 min, during which the total distance covered was recorded and measured. The test apparatus was meticulously cleaned using 75% alcohol, ensuring a pristine environment, prior to each behavioral experiment involving the mice.

#### Novel objective recognition test

The test apparatus is a standard open-field plastic container of 50 × 50 × 50 cm. On the first day, mice were placed in the container for 5 min to acclimate to the environment. Then, the mice were returned to the arena and were given 5 min to familiarize themselves with objects A and B placed at different corners of the cube. After each mouse’s familiarization, the arena and objects were sanitized with 75% ethanol. After 24 h, object B was replaced with object C, and the mice were again given 5 min to explore both objects. The novel object preference was calculated by new object exploration time/(new object exploration time + old object exploration time) × 100%.

#### Y-maze test

The Y-maze is tested to reflect short-term spatial memory. The maze configuration is as follows: three arms of equal length categorized as a start arm (A), an additional arm (B), and a novel arm (C). Each pair of adjacent arms forms a 120° angle, with movable partitions at the center and distinct geometric shapes in each arm serving as visual markers for the mice. In the first trial, the mice explore only two arms (the A arm and the B arm) for 10 min with the novel arm blocked. In the second trial, the novel arm (C) was open, the mice free access to all three arms for 5 min. The total duration and number of crossings into the novel arm (C) were recorded.

### Respiratory energy and general behavior monitoring

The Oxymax CLAMS system (Columbus Instruments) was used to measure the energy metabolism and activity of mice for 24 h, then the mice were acclimated in the laboratory for 24 h before the experiment. Each mouse was kept in a separate cage, with sufficient food and water provided. The O_2_ consumption and energy expenditure were recorded in 24 h. The system enables three-dimensional monitoring of mice using infrared beam technology. The weight of mice was measured before the experiment and recorded in the software. Both VO_2_ and VCO_2_ were directly recorded through software. RER (Respiratory Exchange Ratio): The ratio of VCO_2_ to VO_2_, which reflects the current metabolic status of mice. HEAT: The total heat production rate per unit time of the tested mouse in the current state (kcal/hr). Heat = (3.815 + 1.232 × RER) × VO_2_.

### Western blotting

Primary cultured hippocampal astrocytes and neurons, and freshly excised brain tissue were homogenized at 4 °C and centrifuged at 12,000 rpm for 10 min to collect the supernatant. A bicinchoninic acid (BCA) protein kit (Thermo Fisher Scientific) was used to quantify the protein concentration. The proteins were separated by SDS-polyacrylamide gel electrophoresis and then transferred onto a nitrocellulose membrane. After incubating in 5% skim milk at room temperature for 1 h, the membranes were incubated with the primary antibody overnight at 4 °C. The antibodies used are listed in Table 1. The imprints were then incubated with IRDye TM (800 CW) anti-mouse/anti-rabbit IgG or HRP-conjugated goat anti-mouse IgG/anti-rabbit IgG/donkey anti-goat IgG at 25 °C for 1 h. The antibodies used are listed in Table 2. The images were viewed with the Odyssey infrared imaging system (LI-COR Biosciences, USA) or the ChemiScope (Clinx Science Instruments Co. Ltd).

**Table 2:** Secondary antibodies used in this study.

Antibodies	Source	Dilution
CoraLite488-conjugated Goat Anti-Rabbit IgG(H + L)	Proteintech (SA00013-2)	1:300 for IF
CoraLite488-conjugated Goat Anti-Mouse IgG(H + L)	Proteintech (SA00013-1)	1:300 for IF
CoraLite594–conjugated Goat Anti-Rabbit	Proteintech (SA00013-4)	1:300 for IF
CoraLite594–conjugated Goat Anti-Mouse	Proteintech (SA00013-3)	1:300 for IF
Alexa Fluor® 594-conjugated AffiniPure™ Donkey Anti-Goat IgG (H + L)	Jackson (705-585-147)	1:300 for IF
HRP-conjugated Goat anti-Mouse IgG (H + L)	Abclonal (AS003)	1:10,000 for WB
HRP-conjugated Goat anti-Rabbit IgG (H + L)	Abclonal (AS014)	1:10,000 for WB
HRP-conjugated Donkey anti-Goat IgG (H + R)	Abclonal (AS031)	1:10,000 for WB

*WB* Western blotting, *IF* Immunofluorescence.

We isolated primary hippocampal neurons mitochondrial components according to mitochondrial extraction kit (C3601, Beyotime). Primary hippocampal neurons were collected: washed with PBS and digested with Trypsin-EDTA Solution (200 g), centrifuged at room temperature for 10 min to collect cells. Wash cells: Gently suspend cell precipitate with PBS pre-cooled in ice bath, 600 g, centrifuge at 4 °C for 5 min to precipitate cells. Discard supernatant. Pre-treatment: Add 2 mL of mitochondrial separation reagent, gently suspend the cells, and place them in an ice bath for 10 min. Homogenate: Transfer the cell suspension to a glass homogenizer of appropriate size, homogenate about 20 times. Identification of homogenizing effect: After homogenizing 10 times, take about 2 μL cell homogenate, add 50 μL Trypan blue dyeing solution, and observe the proportion of Trypan blue staining positive (blue) cells under the microscope after mixing. If the proportion of positive cells is less than 50%, increase the homogenate 5 times. Then Trypan blue staining was performed with the previous sample. When the positive proportion exceeds 50%, the homogenate can be stopped and proceeded to the next step. Centrifuge the cell homogenate at 600 g and 4 °C for 10 min. The supernatant was transferred to another centrifugal tube and centrifuged at 11,000 g and 4 °C for 10 min. Remove the supernatant and precipitate the isolated cell mitochondria.

### Enzyme-linked immunosorbent (ELISA)

Hippocampal tissues, primary cultured cells, and astrocyte-conditioned medium (ACM) were lysed in RIPA buffer, centrifuged at 3000 × *g* for 10 min at 4 °C, and the supernatant was collected. Anti-mouse IL-1β, IL-6, and LCN2 ELISA kits were used to measure the levels of IL-1β, IL-6, and LCN2, following the manufacturer’s instructions. The mouse IL-1β and IL-6 ELISA kits were obtained from Abclonal Technology (Product Nos. RK04878, RK00008), and the LCN2 ELISA kit was acquired from Proteintech (Product No. KE10045).

### Primary hippocampal astrocyte culture

C57 mice were anesthetized with isoflurane within 24 h of birth. The hippocampus was quickly removed and placed in Hank’s buffered saline solution and rinsed away the surface blood. The hippocampus was dissected and lightly chopped in Hank’s buffered saline solution, then suspended in 0.25% (v/v) trypsin solution for 9 min at 37 °C. The enzyme reaction was terminated by adding a 1:1 culture solution (Cell Medium + 10% FBS + 1% P/S), then the cells were inoculated into a culture bottle and incubated at 37 °C in a 5% CO2 incubator for 1 week. Sorting Astrocytes: the culture bottles were placed on a shaker at 37 °C for 5 h at 180 RPM. The supernatant was discarded, and the culture bottle was gently rinsed with Hank’s buffered saline solution. The cells were digested with trypsin solution for 3 min, then the culture medium (Cell Medium + 10% FBS + 1% P/S) was added to resuspend the cells. Finally, the cells were plated on dishes coated with 100 μg/mL poly-D-lysine. The astrocytes were treated with LPS or AAV-GFP-vector/AAV-GFP-Sh-LCN2 as required by the experiment.

For preparation of ACM, confluent cultures of astrocytes in 10 cm dishes were washed three times in PBS and fed with 10 mL hippocampal neurons medium. ACM was harvested after 3 days of conditioning and filtered through a 0.2 μm syringe filter. These conditioned media were centrifuged at 1000 g for 10 min at 4 °C and the supernatants were used as ACM. Hippocampal neurons were cultured for 5 days to allow robust process outgrowth and then cultured with ACM for an additional 4 days.

### Primary hippocampal neuron culture

Primary hippocampal neurons were isolated from 17- to 18-day-old C57 mice embryos. The hippocampus was dissected, finely chopped in Hank’s buffered saline solution, and incubated in 0.25% trypsin solution for 9 min at 37 °C. Cells were seeded into 6-well and 12-well plates precoated with 100 μg/mL poly-D-lysine, and the medium was supplemented with 2% B-27 and 1x GlutaMAX. On the third day of culture, ARA-C was added to the basal medium for 24 h at a concentration of 5 mmol/L to eliminate glial cells. The basal medium was subsequently replaced with fresh medium containing B-27 every 3 days. The neurons were treated with recombinant LCN2 protein and ACM as required by the experiment. The medium was replaced with ACM on DIV5. The effects of ACM on dendritic elongation were assayed on DIV9. Cells were collected and lysed in RIPA buffer for bioassays or fixed with 4% paraformaldehyde for immunofluorescence imaging. Sholl analysis involves using the cell body as the center of concentric circles and counting the intersection points between dendritic branches and circles of increasing radii to quantify the complexity of dendritic arborization. Dendritic length analysis was conducted using ImageJ software.

### Recording of long-term potentiation (LTP)

The mice’s brains were rapidly excised by a vibrating microtome (Leica, Wetzlar, Germany) at a thickness of 300 μm and perfused with 30 ml of ice-cold aCSF solution containing the following (in mM): 120 NaCl, 2.5 KCl, 2 CaCl_2_·2H_2_O, 1.25 KH_2_PO4, 2 MgSO4·7H2O, 26 NaHCO_3_, and 10 glucose, saturated with 95% O_2_ and 5% CO_2_ and buffered to a pH of 7.4. The slices were continuously perfused with oxygenated aCSF. For LTP measurement, the slices were placed in a chamber equipped with an 8 × 8 microelectrode array (Parker Technology, Beijing, China), with each electrode measuring 50 × 50 μm, and immersed in aCSF. The stimulus signal was provided by the MED64 system (Alpha MED Sciences, Panasonic). and the fEPSPs of DG-CA1 neurons were recorded following three series of high-frequency stimulation (HFS; 100 Hz, 1-s duration) by stimulating DG neurons. The magnitude of LTP was quantified by the fEPSP slope over 1 h.

### Golgi staining

Preparation of Golgi solution: A: 5% potassium dichromate (10 g of potassium dichromate were dissolved in 200 mL ddH_2_O); B: 5% mercury chloride (10 g of mercury chloride were dissolved in 200 mL ddH_2_O); 5% potassium chromate (8 g of potassium chromate were dissolved in 160 mL ddH_2_O). 5% potassium dichromate and 5% mercury chloride were mixed to form the AB solution; 400 mL ddH_2_O was mixed with solution C; Finally, solution AB was added to solution C (prepared in a 1000 mL large beaker). The volumetric ratio of the prepared Golgi solution is potassium dichromate: mercuric chloride: potassium chromate: water = 5:5:4:10. Place in the dark for about a week to allow impurities to precipitate.

Procedure: mice were anesthetized with isoflurane, about 300 mL of normal saline was injected into the left ventricle, and the brain tissue was taken out and soaked in Golgi solution in the dark for 30 days. The Golgi solution was changed every 2 days. The brain was sliced using a vibrating microtome (Leica, Wetzlar, Germany) at a thickness of 100 μm. Staining: alkalization with ammonia, dehydration, and transparent soaking in CXA solution for 15 min (1000 mL chloroform + 1000 mL xylene + 1000 mL anhydrous ethanol, 1:1:1), sealed with neutral gum and dried. Image Analysis: Sholl analysis (using the cell body as the center of concentric circles and counting the intersection points with circles of different radii to reflect the number of dendritic branches). In ImageJ software, the unit dendrite length was marked, and the software automatically recorded the number of dendritic spines.

### Nissl staining

Nissl staining solution (C0117, Beyotime) was used to stain the frozen sections for 3 min, then wash distilled water 2 times for 1 min and dehydrate with 75%, 85%, and 95% ethanol for 2 min. The slides were cleaned twice with xylene for 5 min each, seal the brain slice with a neutral gum sealant. The images were visualized using the automatic Slice Scanning System (VS200, Olympus, Japan). Cell numbers were analyzed using ImageJ software.

### TUNEL assay

Cellular DNA fragmentation in primary hippocampal neurons was determined by the One Step TUNEL Apoptosis Assay Kit (Dalian Meilun Biotechnology Co., Ltd., Product ID: MA0224). Perform experimental procedures according to the instructions, neurons were fixed in 4% paraformaldehyde for 30 min, then permeabilized in 0.5% Triton X-100 in PBS for 15 min. Nuclear staining was performed using DAPI. The images were captured using a Zeiss LSM 710 laser scanning confocal microscope (Zeiss, Jena, Germany). The apoptosis index was calculated by colocalization of Tunel and DAPI positive cell/ DAPI positive cell ×100%.

### Fluorescence imaging and confocal microscopy

Brain slices and primary hippocampal astrocytes/neurons were fixed with 4% paraformaldehyde and permeabilized at room temperature in PBS with 0.5% Triton X-100 for 0.5 h. Wash three times by PBS for 10 min each time. Both brain slices and primary astrocytes/neurons were then blocked using 5% bovine serum albumin (BSA) for 1 h. They were subsequently incubated with the primary antibody overnight at 4 °C, washed three times by PBS for 10 min each time, followed by incubation with CoraLite488 and 594-conjugated secondary antibodies at 37 °C for 1 h. The antibodies used are listed in Table 2. Nuclear staining was performed using DAPI. All fluorescence images were captured using a Zeiss LSM 710 laser scanning confocal microscope (Zeiss, Jena, Germany) or automatic Slice Scanning System (VS200, Olympus, Japan). ImageJ software was used for image analysis. The fluorescence intensity was analyzed using ImageJ software. Colocalization was quantified using the Coloc 2 plugin in ImageJ. The relative fluorescence intensity of LCN2 in astrocytes was calculated by colocalization of LCN2 and GFAP/GFAP fluorescence intensity. The relative fluorescence intensity of LCN2 in microglia was calculated by colocalization of LCN2 and iba1/iba1 fluorescence intensity.

### ATP assay

The ATP assay kit (S0026 and S0027, Beyotime) is developed by using firefly luciferase to catalyze luciferin and produce fluorescence, which requires ATP to provide energy. The fluorescence production is proportional to ATP concentration within a concentration range from 0.1 nM to 10 µM, which allows for highly sensitive detection of ATP concentration. Brain tissues and primary hippocampal neurons were lysed using the lysis buffer provided in the ATP assay kit. The standardized sample protein was added to 100 μL of ATP detection buffer to measure ATP production by the manufacturer’s instructions. The resulting luminescence was quantified by using a luminometer. ATP production was calculated.

### SOD activity assay

Total SOD activity in hippocampal tissues and primary hippocampal neurons was assessed using a Cu/Zn-SOD and Mn-SOD Assay Kit (S0103, Beyotime). The Cu/Zn-SOD and Mn-SOD Assay Kit with WST-8 is a colorimetric assay kit for the determination of Cu/Zn-SOD, Mn-SOD or total SOD activity in cells, tissues or other samples based on WST-8. Brain tissues and primary hippocampal neurons were prepared by the manufacturer’s instructions. The standardized sample protein was added to 160 μL of WST-8/ Enzyme working fluid, use 96-well plates to set up sample holes, add the sample to be tested and other solutions in turn, add the reaction start-up working liquid and mix thoroughly, incubate at 37 °C for 30 min. A microplate reader was used to measure the absorbance of each sample at 450 nm.

### MDA assay

The MDA levels in hippocampal tissues and primary hippocampal neurons were measured using the Lipid Peroxidation MDA Assay Kit (S0131S, Beyotime). Lipid Peroxidation MDA Assay Kit uses a color reaction based on the reaction of MDA and thiobarbituric acid (TBA) to produce red product. Then colorimetry was used to quantitatively detect MDA in tissue or cell lysate. The hippocampal tissues and primary hippocampal neurons were obtained and lysed. The standardized sample protein was added to a 96-well plate according to the instructions, and the absorbance of the solution was measured at a wavelength of 532 nm.

### DHE staining

Dihydroethidium (DHE) is one of the most commonly used fluorescent probes for the detection of superoxide anions, which can be dehydrogenated under the action of superoxide anions in cells to produce ethidium. Ethidium can bind to RNA or DNA to produce red fluorescence. When the levels of superoxide anion in the cell are higher, more ethidium is produced, and the red fluorescence is stronger, and vice versa. This allows for the detection of superoxide anion levels with dihydroethidium. Brain slices were immersed in a solution containing 5 μM fluorescent dye DHE (S0063, Beyotime) and kept in the dark at 37 °C for 30 min, then the slices were washed three times with PBS. Images were captured using automatic Slice Scanning System (VS200, Olympus, Japan). The fluorescence intensity was analyzed using ImageJ software.

### Datasets collection and processing

In this study, bioinformatics analyses were conducted to identify marker genes associated with LPS models and the function of LCN2 exposure in neuron [17]. The datasets GSE88959 [62] were obtained from the Gene Expression Omnibus (GEO) database. Corresponding mRNA expression matrices were aligned for comprehensive analysis.

In the GSE88959 dataset, an RNAseq transcriptomic analysis was performed from PBS perfused hemi-brain samples after 8 h post peripheral administration of LPS. Three- to four-month-old C57BL/6J mice of both sexes were used. As no sex-based differences were observed, data were combined for analysis. For LPS administration, mice received an intraperitoneal injection of 2 μg/g body weight. The control group consisted of samples from GSM2356404 to GSM2356407, serving as the reference for bioinformatics analysis. The experimental group, representing the LPS models, included samples from GSM2356412 to GSM2356415, which were exposed to LPS for 8 h. The mRNA expression matrices from these samples were normalized using the “limma” R package. Additionally, Differential Expression Genes (DEGs) of the LCN2-treated primary hippocampal neurons were obtained from National Center for Biotechnology Information (NCBI) [17].

### Bioinformatics analysis

Differential Expression Genes (DEGs) were identified using the “limma” R package with criteria set at |Log2FoldChange| > 1 and P < 0.05. Gene Ontology (GO) enrichment analysis and Kyoto Encyclopedia of Genes and Genomes (KEGG) pathway enrichment analysis were conducted using the “biomaRt” and “clusterProfiler” R packages, with a significance threshold of p-value < 0.05 for the enrichment terms. Visualization of KEGG and GO enrichment analysis results was performed using the “aPEAR”, “GOplot”, and “ggplot2” R packages. Venn diagrams illustrated the intersection of |Log2FoldChange| > 2 of DEGs with core genes in “Immune system” pathways from Reactome Pathway Database. Protein-Protein Interaction (PPI) networks were constructed using the brain network (edge weight > 0.5) from the TissueNexus online database. The top 5 hub genes were identified through DMNC analysis using the MCODE and Cytohubba plugin in Cytoscape 3.10.2 software. Enrichment analysis of different cell death was conducted using “GSVA” R packages and XDeathDB database. Different metabolism pathways enrichment analysis was conducted using “GSVA” R packages.

### Statistical analysis

In vitro assays, the experiments were conducted by a blind individual, who did not know the sample information. The animal’s information for vivo assays was decoded after all samples were analyzed. Sample size was determined by Power and Precision (Biostat). Data were presented as the mean ± SEM and analyzed using GraphPad Prism 8.0 statistical software (https://www.graphpad.com/scientific-software/prism/). A two-tailed Student’s T-test was used to assess the variance between two groups, and the differences among multiple groups were determined using one- or two-way analysis of variance (ANOVA). P < 0.05 was considered statistically significant.

## Supplementary information

Supplementary material

wb-Original banding20250105

## Data Availability

The datasets used and/or analyzed during the present study are available from the corresponding author upon reasonable request.
