# Supplementary material for: LCN2 induces neuronal loss and facilitates sepsis-associated cognitive impairments
Source: Cell Death Dis. 2025 Mar 1;16(1):146. doi: 10.1038/s41419-025-07469-4 (PMC11873032; doi:10.1038/s41419-025-07469-4)
Supplement: Supplementary file 1 — Supplementary material [file 41419_2025_7469_MOESM1_ESM.docx]

**Supplementary Figure 1**


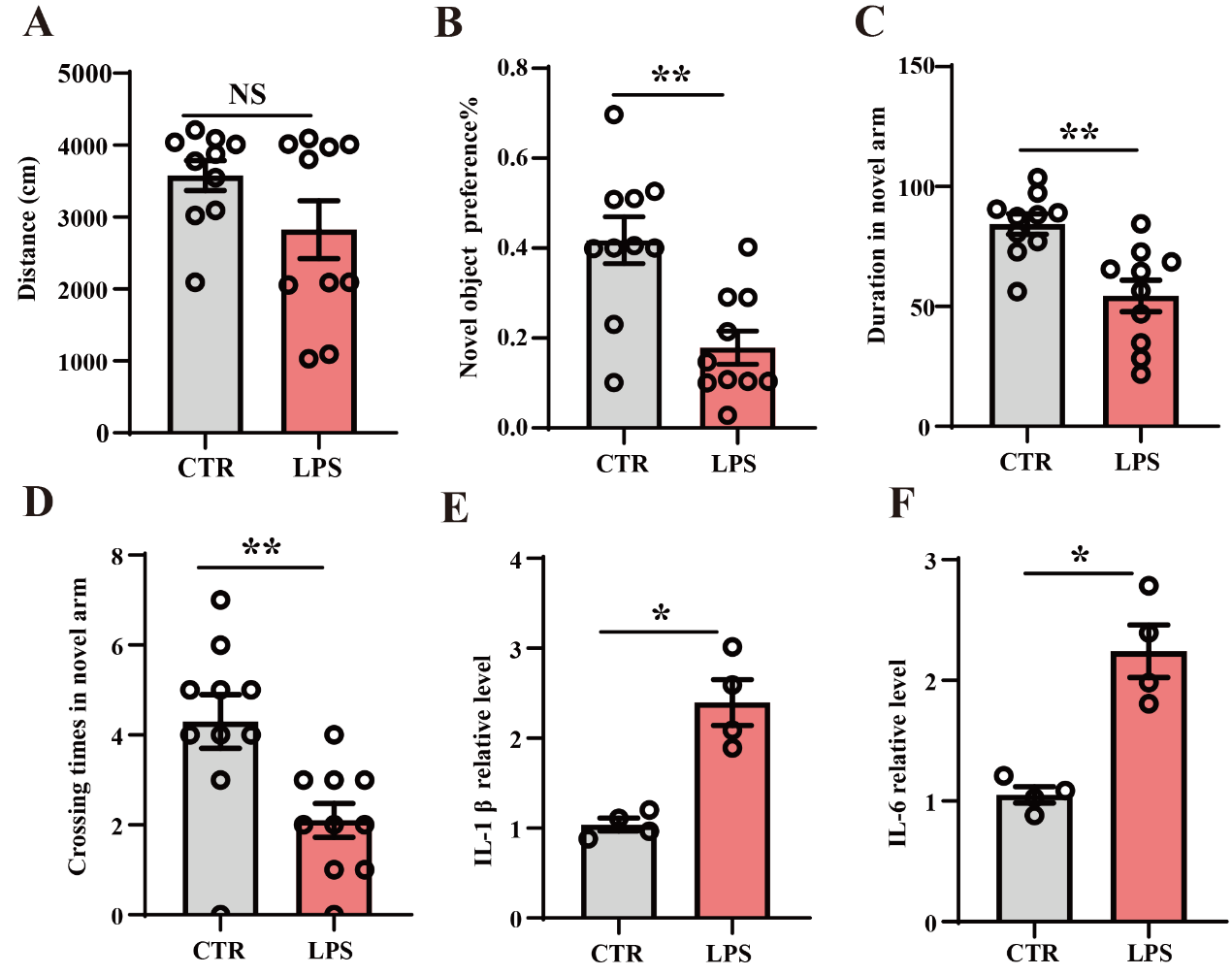


**sFigure 1 LPS induced cognitive impairments, accompanied by neuroinflammatory activation**

The LPS model group and control group were respectively intraperitoneally injected with LPS (10mg/Kg) or normal saline. (A) The open field test was measured to the total distance covered in the two groups (n=10). (B) The novel object recognition test measured the new object preference (n=10). (C, D) Y-mase test: the duration in the novel arm (C) and the crossing number (D) of the novel arm were measured (n=10). (E, F) ELISA kits were used to measure the IL-1β (E) and IL-6 (F) (n=4). Data are presented as Mean ± SEM. A two-tailed Student’s t-test was used for statistical analysis in part A, B, C, D. A non-parametric test of Mann-Whitey test was used for statistical analysis in part E, F. *p < 0.05, **p < 0.01, versus control group.

**Supplementary Figure 2**


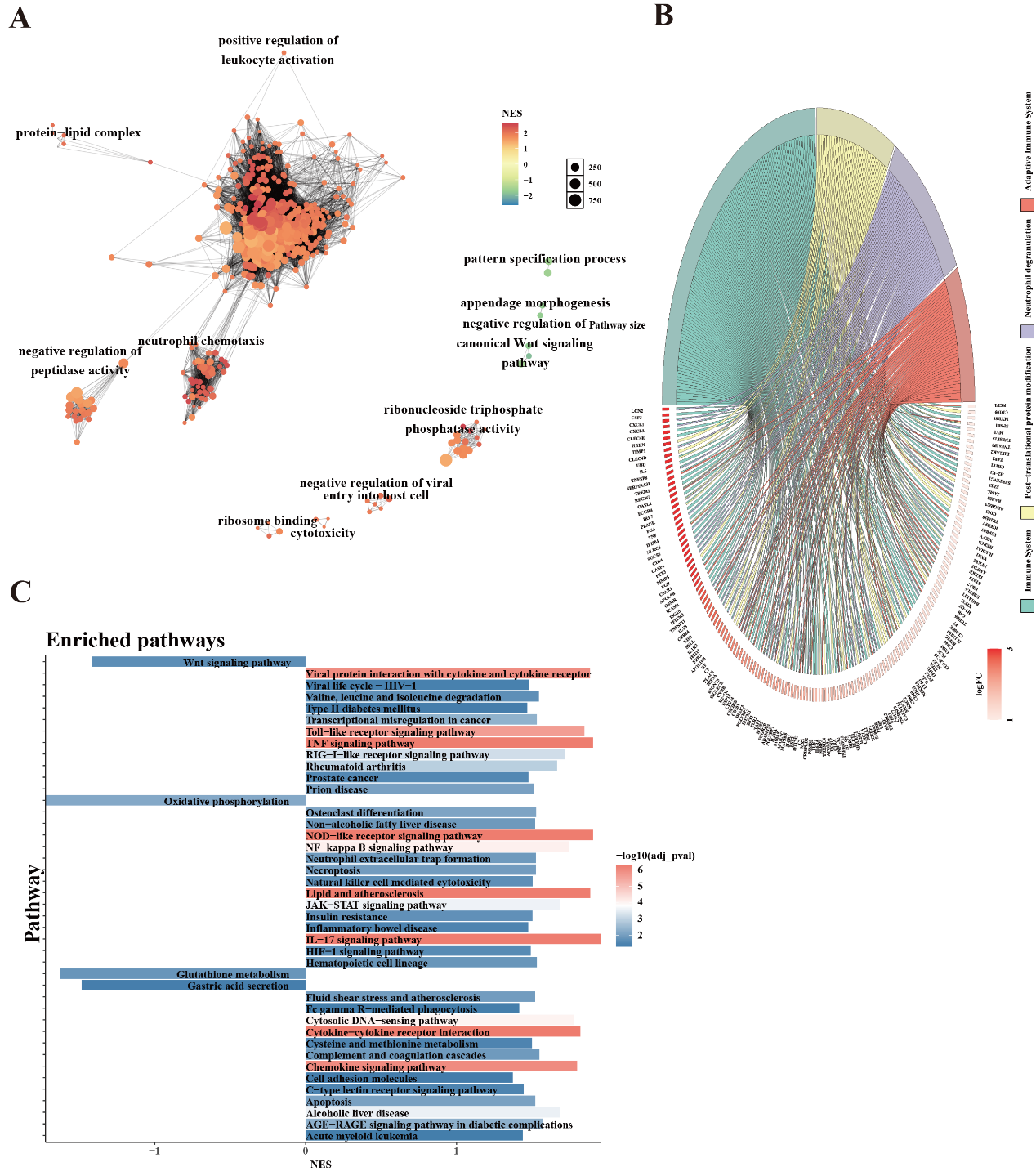


**sFigure 2 The visualization of GSE88959 gene expression analysis results**

(A)Gene Ontology (GO) enrichment analysis results ­­­for DEGs in GSE88959 DEGs. (B) Visualization of selected Reactome pathways and their core enrichment genes in GSE88959. (C) KEGG pathways enriched in GSE88959 DEGs.

**Supplementary Figure 3**


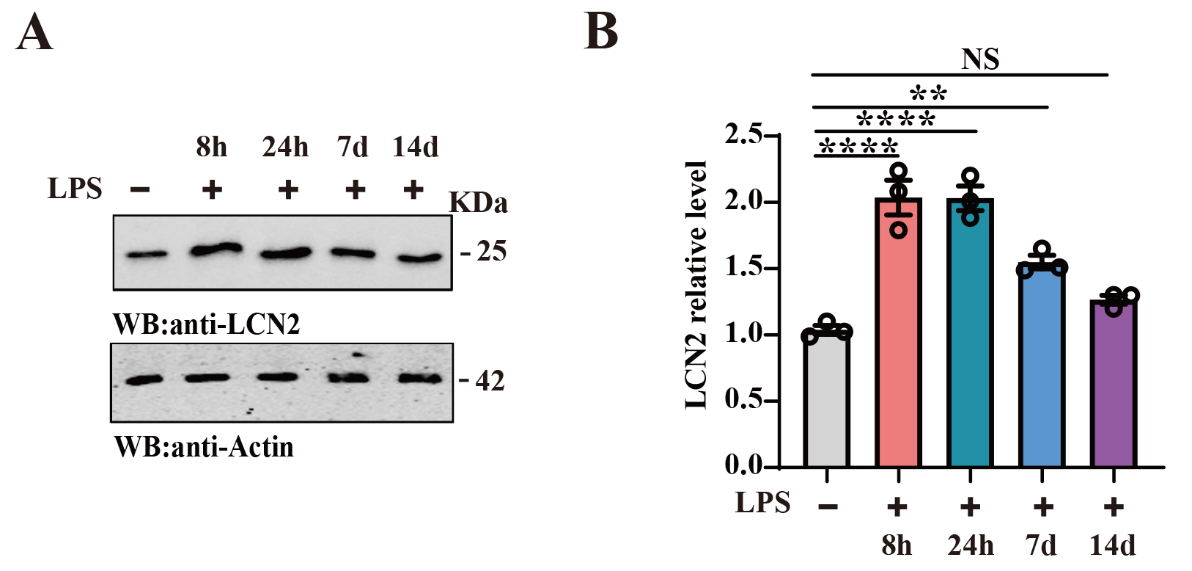


**sFigure 3** **LCN2 levels were increased in the hippocampus** **after intraperitoneal injection of LPS 1 week.**

(A) LCN2 levels in the hippocampus were measured 8 hours, 24 hours, 7 days, and 14 days after intraperitoneal injection of LPS. Brain tissues (hippocampus) were homogenized and LCN2 levels were detected by immunoblotting. Actin was used as a loading control. (B) Quantitative analysis of the LCN2, (n=3). Data are presented as Mean ± SEM. one-way ANOVA was used for statistical analysis. **p < 0.01, ****p < 0.0001, versus Control group.

**Supplementary Figure 4**


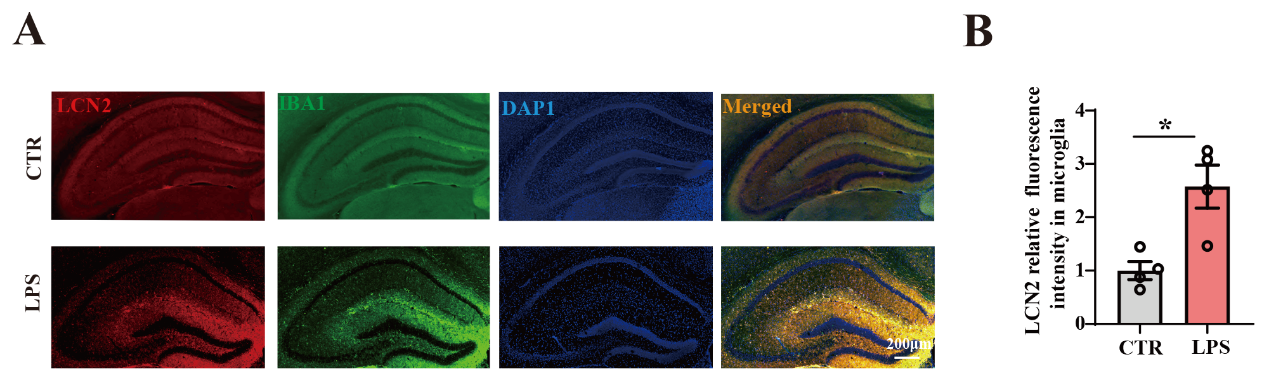


**sFigure 4 LCN2 levels were increased in the hippocampus after intraperitoneal injection of LPS.**

(A)Representative immunofluorescence images show IBA1 and LCN2 fluorescence in brain slices of two groups. (B) Quantitative analysis of IBA1 and LCN2 fluorescence in hippocampal slices, (n=4 slices from 4 mice). Data are presented as Mean ± SEM. A non-parametric test was used for statistical analysis. *p < 0.05, versus Control group.

**Supplementary Figure 5**


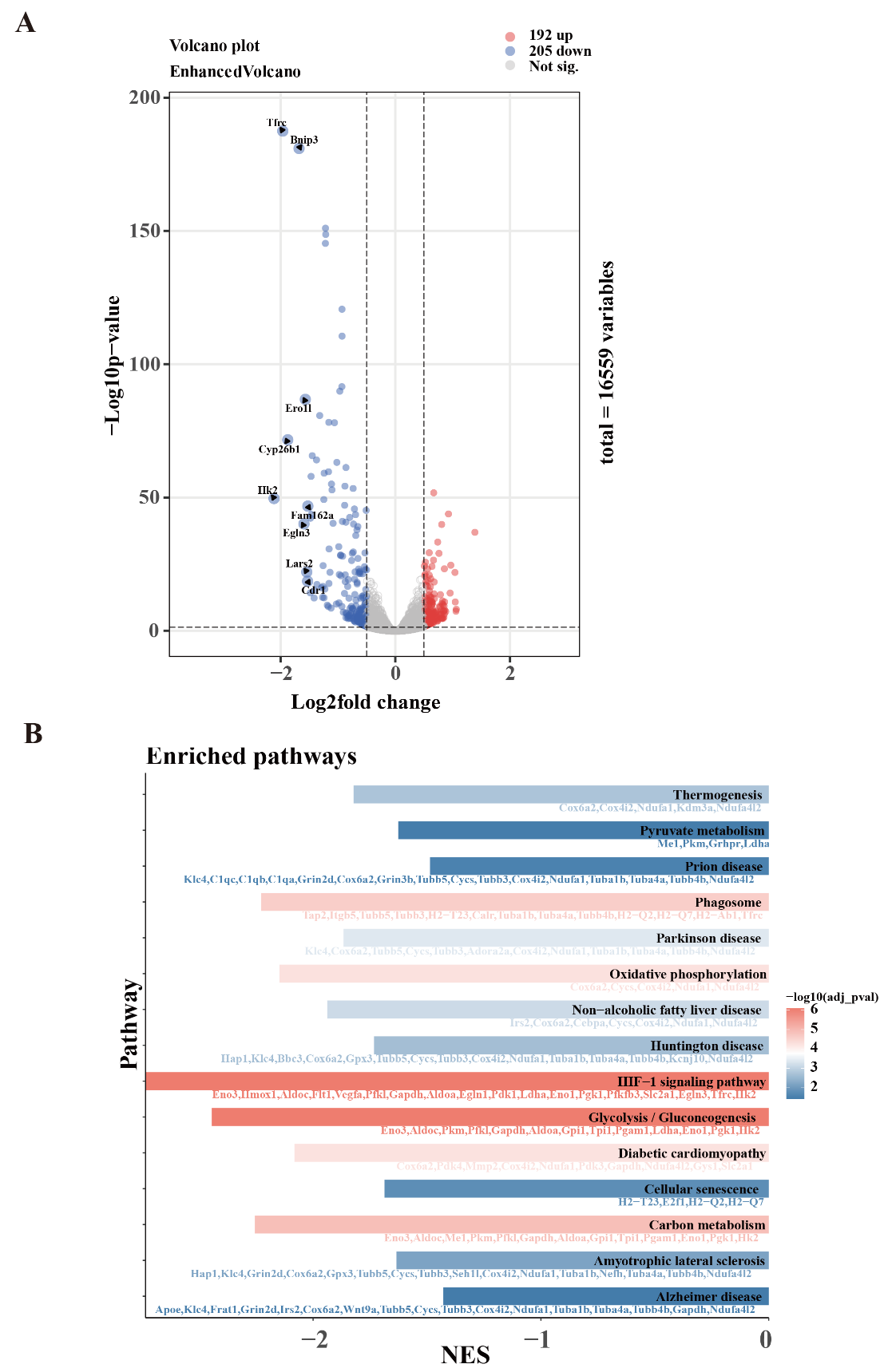


**sFigure 5 The visualization of LCN2-treated primary hippocampal neurons gene expression analysis results**

(A) Volcano plot of DEGs from LCN2-treated primary hippocampal neurons. Genes with |Log2FoldChange| > 1.5 are highlighted. (B)KEGG pathways enriched in DEGs from LCN2-treated primary hippocampal neurons.

**Supplementary Figure 6**

**
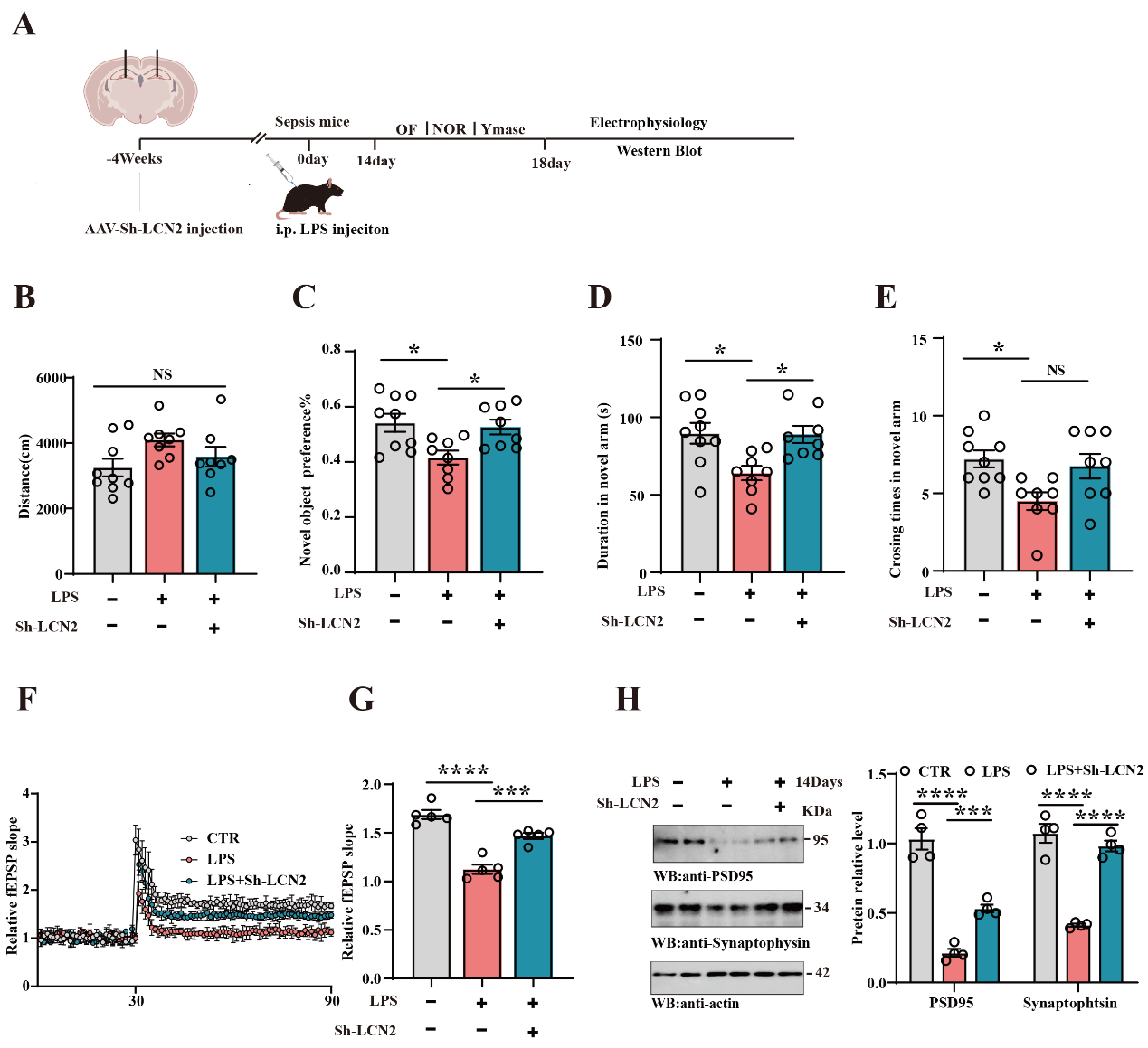
sFigure 6** **Downregulation of LCN2 alleviated sepsis-related synaptic and cognitive impairments 2 weeks after intraperitoneal injection of LPS.**

(A) Experimental design sketch. In the control group, mice were bilaterally injected with AAV-Sh-vector in the DG region and injected intraperitoneally with normal saline. In the model group, mice were bilaterally injected with AAV-Sh-vector in the DG region and injected intraperitoneally with LPS. In the preventive group, mice were bilaterally injected with AAV-Sh-LCN2 in the DG region and received intraperitoneal injections of LPS. Following the treatment, behavioral, electrophysiological, and biochemical tests were performed 2 weeks after intraperitoneal injection of LPS. (B) The open field test measured the total distance covered, (n=8-9). (C) The novel object recognition test measured the preference, (n=8-9). Y-mase test: the duration (D) and crossing times (E) in the novel arm were measured, (n=8-9). (F) Hippocampal DG-CA1 Long-Term Potentiation was recorded using the MED64 system. (G)The normalized mean slope of fEPSP was recorded in hippocampal slices, (n=5 slices from 5 mice). (H) Brain tissues (hippocampus) from the three groups were homogenized and synaptic associated protein were detected by immunoblotting, actin was used as a loading control. Quantitative analysis of the PSD95, Synaptophysin, (n=4). Data are presented as Mean ± SEM, one-way or two-way ANOVA was used for statistical analysis. *p < 0.05, ***p < 0.001, ****p < 0.0001, versus LPS group.
