# Supplementary material for: LCN2 induces neuronal loss and facilitates sepsis-associated cognitive impairments
Source: Cell Death Dis. 2025 Mar 1;16(1):146. doi: 10.1038/s41419-025-07469-4 (PMC11873032; doi:10.1038/s41419-025-07469-4)
Supplement: Supplementary file 2 — wb-Original banding20250105 [file 41419_2025_7469_MOESM2_ESM.pptx]

## Slide 1
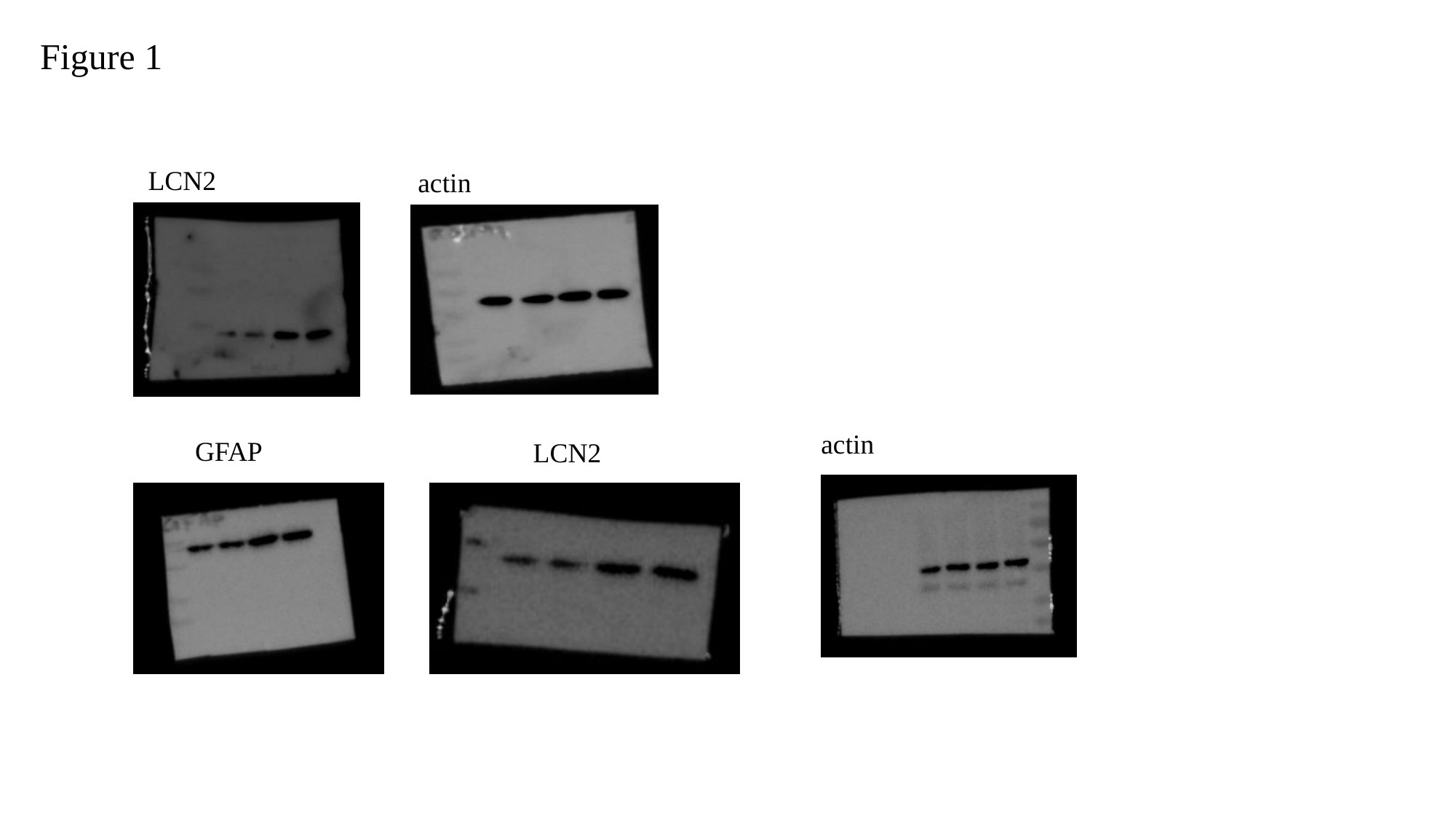

Figure 1
LCN2
actin
actin
GFAP
LCN2

## Slide 2
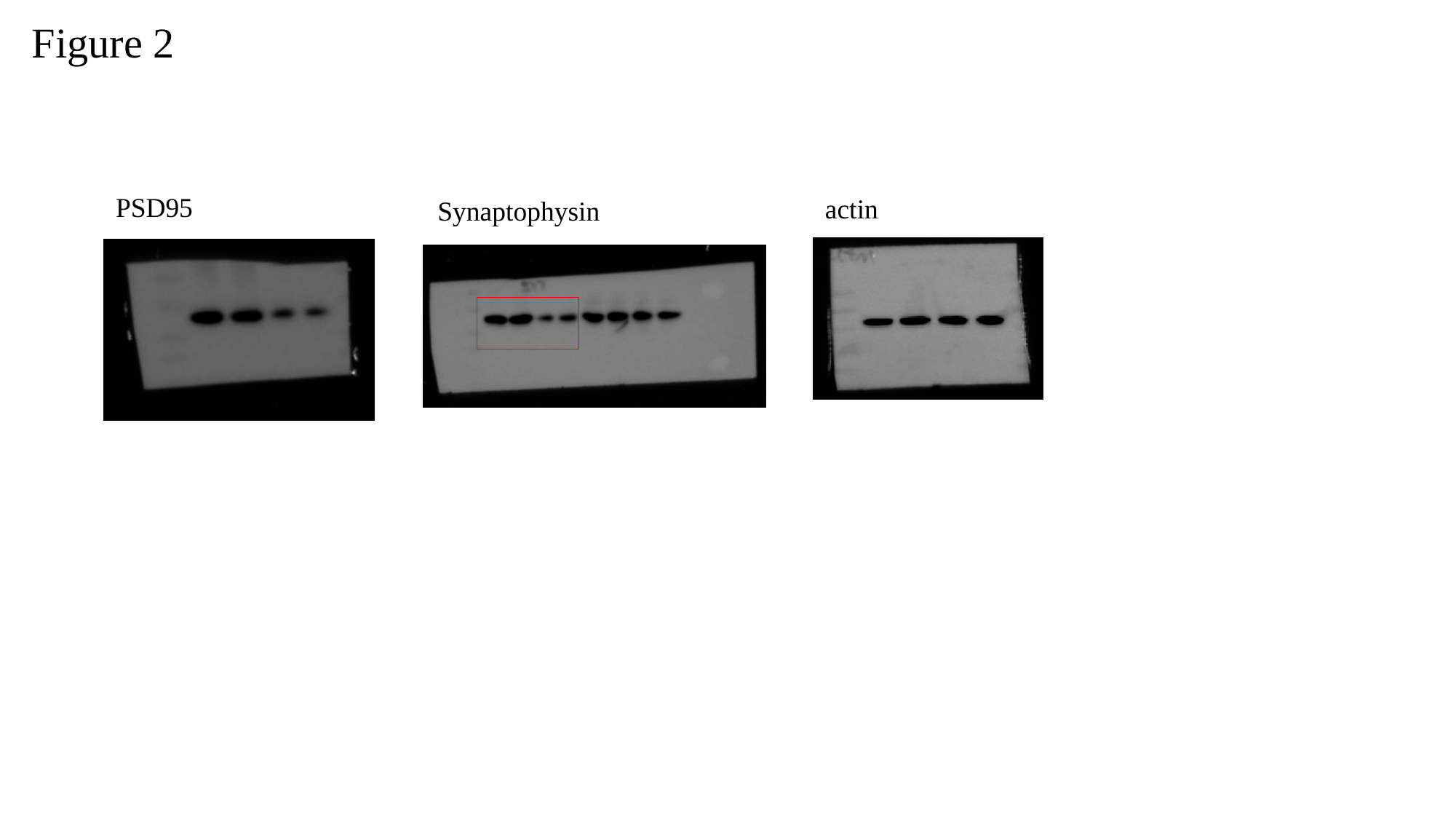

Figure 2
PSD95
actin
Synaptophysin

## Slide 3
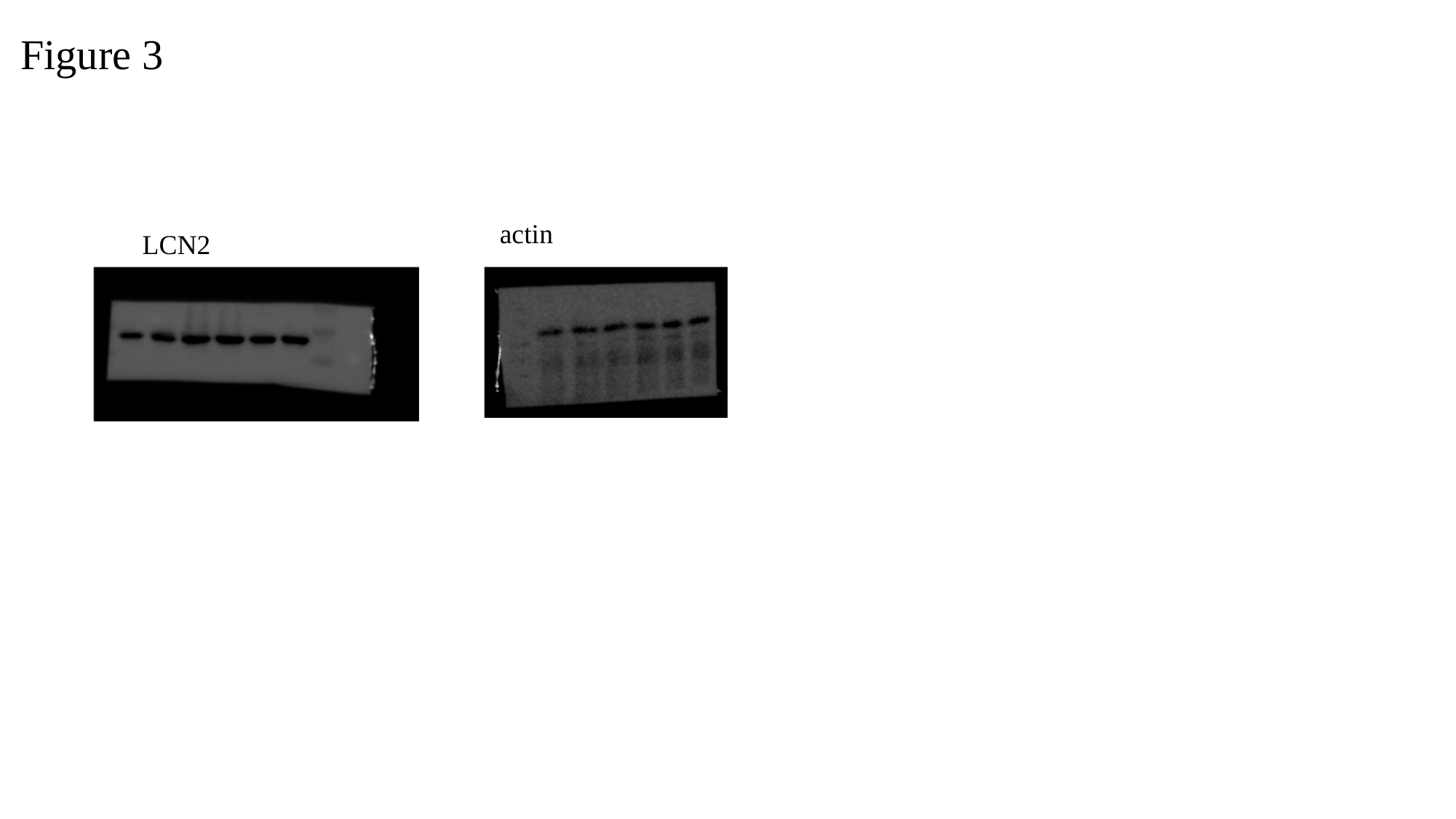

Figure 3
actin
LCN2

## Slide 4
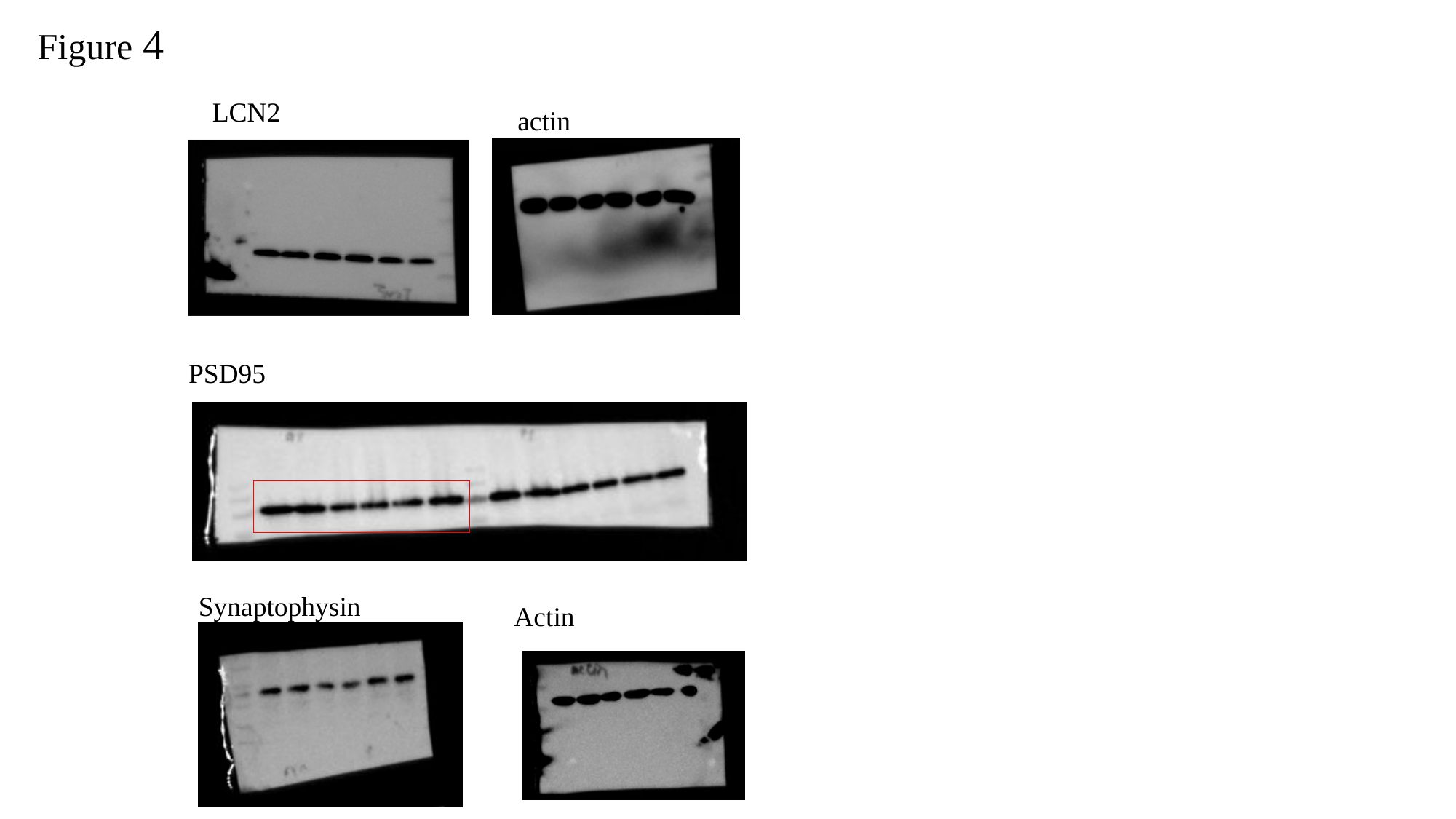

Figure 4
LCN2
actin
PSD95
Synaptophysin
Actin

## Slide 5
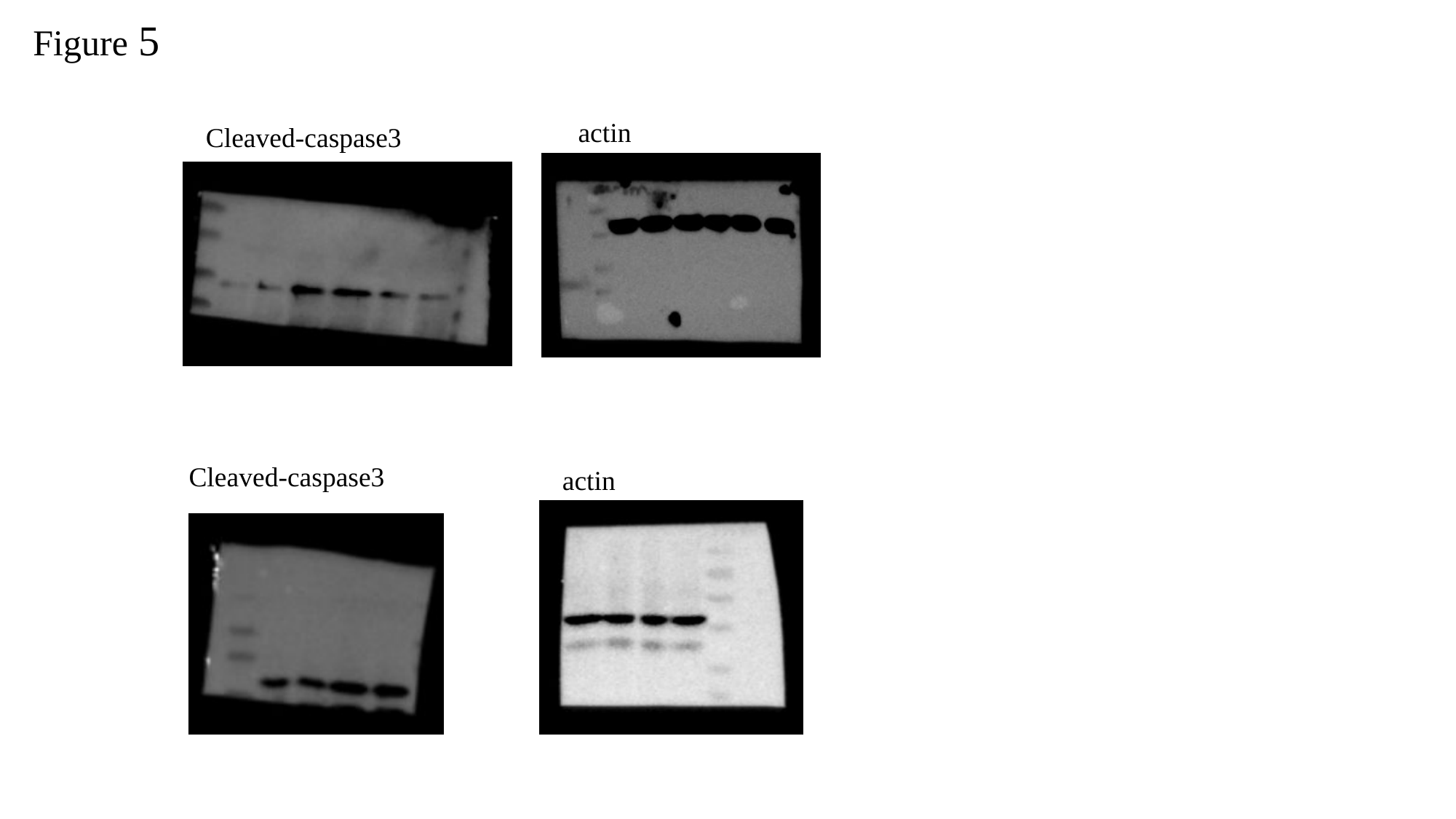

Figure 5
actin
Cleaved-caspase3
Cleaved-caspase3
actin

## Slide 6
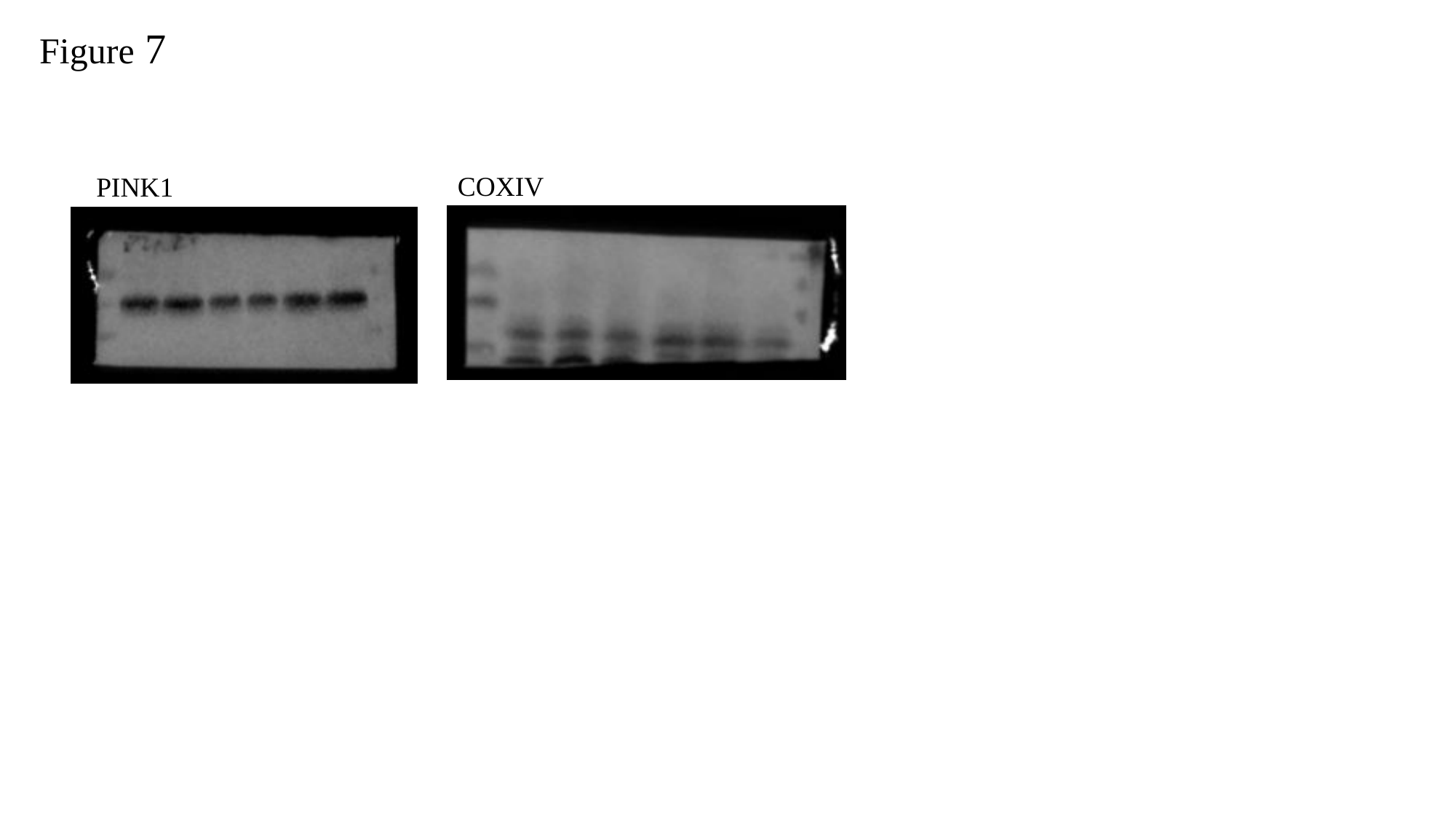

Figure 7
COXIV
PINK1

## Slide 7
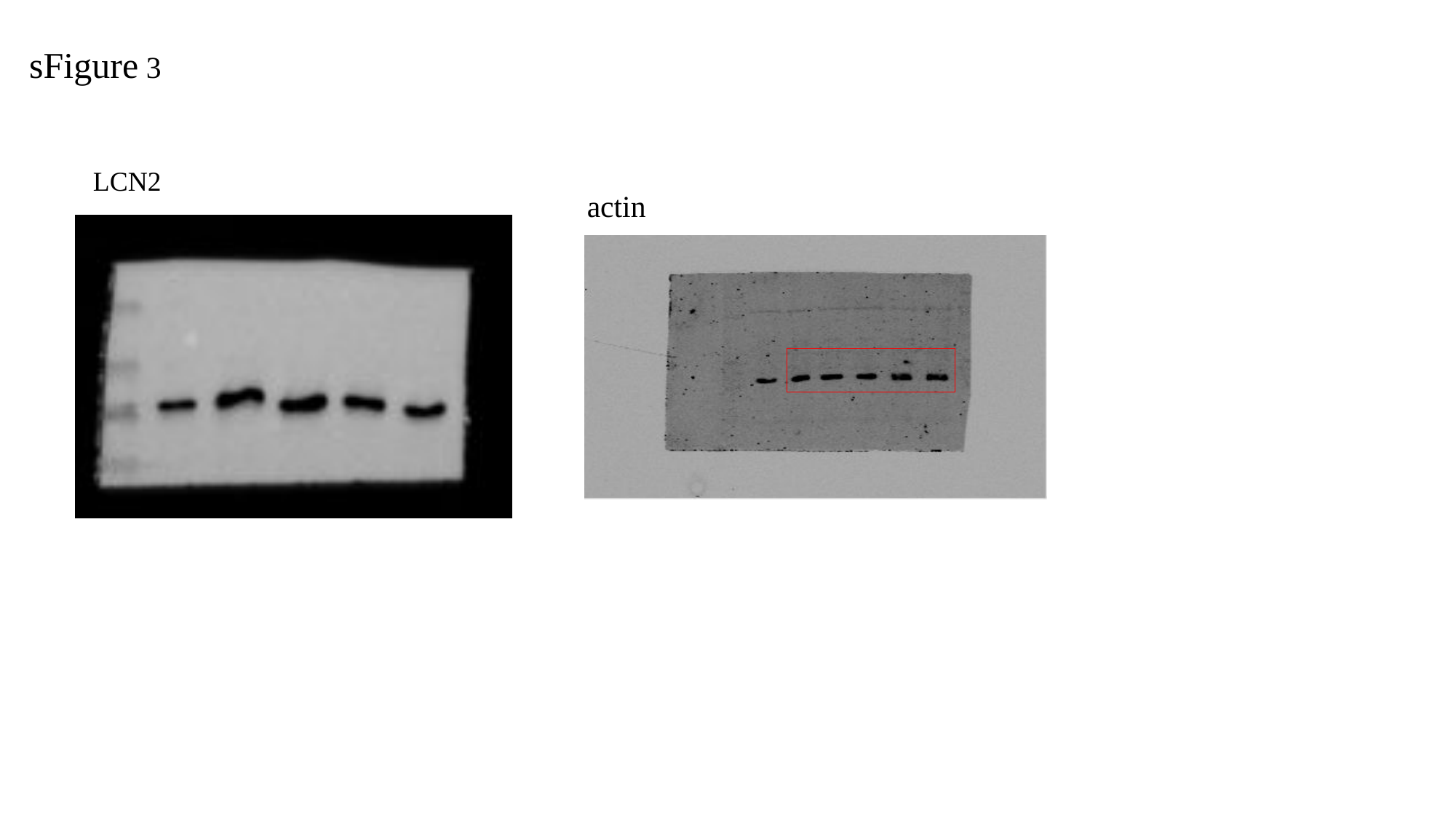

sFigure 3
LCN2
actin

## Slide 8
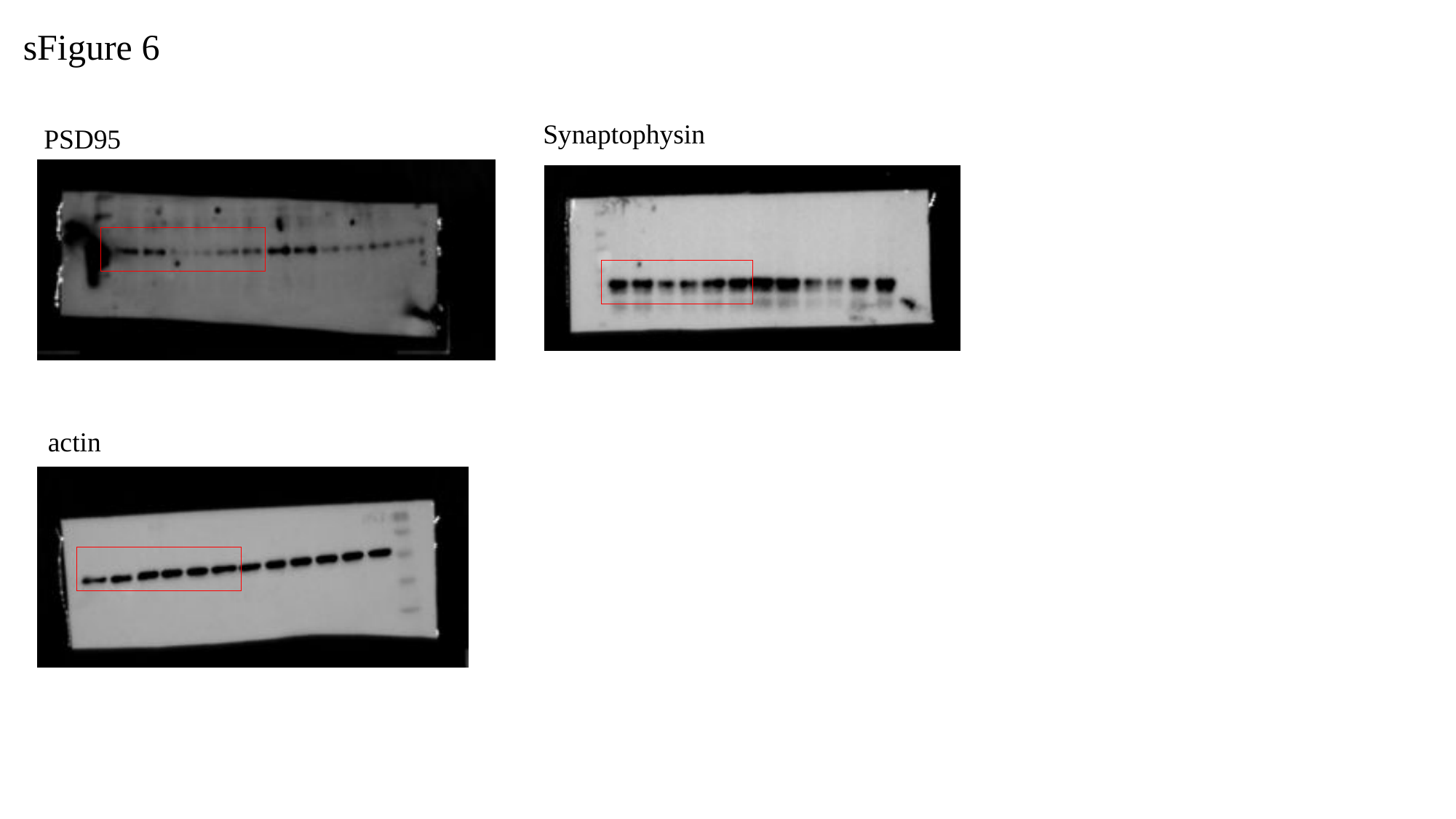

sFigure 6
Synaptophysin
PSD95
actin
